# A Model for the Prediction of Mortality and Hospitalization in Chinese Heart Failure Patients

**DOI:** 10.3389/fcvm.2021.761605

**Published:** 2021-11-18

**Authors:** Bo Zhuang, Ting Shen, Dejie Li, Yumei Jiang, Guanghe Li, Qian Luo, Yishan Jin, Ziwei Shan, Lin Che, Lemin Wang, Liang Zheng, Yuqin Shen

**Affiliations:** ^1^Department of Rehabilitation, Tongji Hospital Affiliated to Tongji University, Tongji University School of Medicine, Shanghai, China; ^2^Department of Cardiology, Tongji Hospital Affiliated to Tongji University, Tongji University School of Medicine, Shanghai, China; ^3^Department of Cardiovascular Medicine, Research Center for Translational Medicine, Shanghai East Hospital, Tongji University School of Medicine, Shanghai, China

**Keywords:** risk prediction, mortality, hospitalization, heart failure, Chinese population

## Abstract

**Background:** Although many risk prediction models have been released internationally, the application of these models in the Chinese population still has some limitations.

**Aims:** The purpose of the study was to establish a heart failure (HF) prognosis model suitable for the Chinese population.

**Methods:** According to the inclusion criteria, we included patients with chronic heart failure (CHF) who were admitted to the Department of Cardiac Rehabilitation of Tongji Hospital from March 2007 to December 2018, recorded each patient's condition and followed up on the patient's re-admission and death. All data sets were randomly divided into derivation and validation cohorts in a ratio of 7/3. Least absolute shrinkage and selection operator regression and Cox regression were used to screen independent predictors; a nomogram chart scoring model was constructed and validated.

**Results:** A total of 547 patients were recruited in this cohort, and the median follow-up time was 519 days. The independent predictors screened out by the derivation cohort included age, atrial fibrillation (AF), percutaneous coronary intervention (PCI), diabetes mellitus (DM), peak oxygen uptake (peak VO_2_), heart rate at the 8th minute after the cardiopulmonary exercise peaked (HR8min), C-reaction protein(CRP), and uric acid (UA). The C indexes values of the derivation and the validation cohorts were 0.69 and 0.62, respectively, and the calibration curves indicate that the model's predictions were in good agreement with the actual observations.

**Conclusions:** We have developed and validated a multiple Cox regression model to predict long-term mortality and readmission risk of Chinese patients with CHF.

**Registration Number:** ChicTR-TRC-00000235.

## Introduction

Heart failure (HF) is a serious manifestation of the late stage of various heart diseases. Its morbidity has an increasing trend, and the mortality and rehospitalization rates remain high, posing a huge economic burden for health care systems (1–3). According to recent studies, the 1-year mortality rate of patients with chronic heart failure (CHF) is 6.4%, and the combined death or HF hospitalization rate within 1 year is 14.5% (4). Therefore, stratifying patients according to the risk of future results (re-admission and death), and optimizing treatment strategies for patients with different needs would be beneficial to all healthcare systems and patients.

The 2019 American College of Cardiology (ACC) Expert Consensus on HF proposes that the assessment of risk-increasing factors can help guide decision-making for preventive intervention (5). Many risk prediction models have been released internationally (6–10). The 2017 A systems Biology Study to Tailored Treatment in Chronic Heart Failure (BIOSTAT-CHF) research plan included 2,516 HF patients from 69 centers in 11 international centers. It concluded that the strongest predictors of mortality are age, urea nitrogen, N-terminal B-type natriuretic peptide, hemoglobin, and beta blockers. The predictors of hospitalization rate are age, hospitalization history for HF, edema, systolic blood pressure, and estimated glomerular filtration rate (6). In 2019, the Korean Acute Heart Failure registry used 12 predictors to establish a risk score that predicts the risk of HF-specific readmission or death at 30 days after discharge (7). The 2020 Prospective Comparison of angiotensin receptor neprilysin inhibitor (ARNI) with angiotensin-converting enzyme inhibitor (ACEI) to Determine Impact on Global Mortality and Morbidity in Heart Failure (PARADIGM-HF) model can accurately predict the morbidity and mortality of ambulatory patients with CHF with reduced ejection fraction at 1 and 2 years (8). It can be seen from the above that the current existing forecasting models have the following advantages: Indicators are easily available and not focusing on a single risk factor. However, the application of these models in the Chinese population still has some limitations. Most of these models use static indicators, which are not sufficient to reflect the overall condition of the patients (6–8, 10, 11). Further, most studies have a short follow-up time and cannot judge the long-term prognosis (7, 10, 11). As there are regional differences in the mortality and rehospitalization rates of patients with CHF and scarce data from the Chinese population, the applicability of the international HF risk prediction model to the Chinese population is controversial (4, 12). Therefore, it is necessary to establish a new HF risk prediction model suitable for the Chinese population.

In this study, we selected individuals with CHF in China as the target population for modeling and conducted relevant cardiopulmonary exercise tests (CPETs) to monitor the respiratory and circulatory parameters of patients under exercise to obtain comprehensive indicators of cardiopulmonary function. According to the literature, CPET parameters are predictors of the prognosis of HF, especially the level of peak oxygen uptake (peak VO_2_) (13–15). Of note, our follow-up time is long (the longest follow-up time is as long as 12 years) which is helpful to assess the long-term prognosis of patients with CHF. Our research combines the experimental indicators of cardiopulmonary exercise to propose a long-term prognosis and readmission model for CHF in the Chinese population, which provides a basis for the treatment of clinical CHF and reveals potential targets for interventions to improve prognosis.

## Methods

### Data Sources and Processing

**Figure 1:**
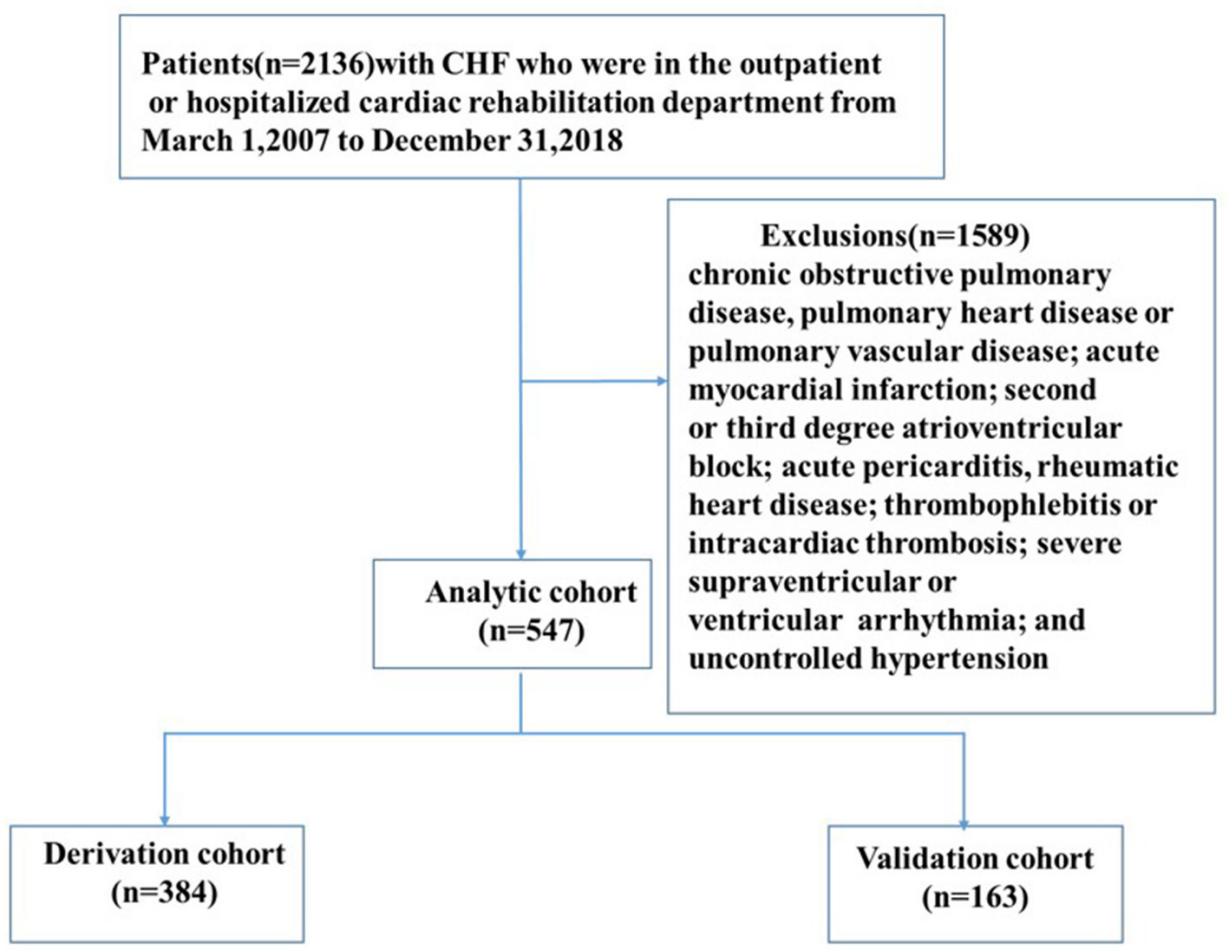
Study flow. CHF, chronic heart failure.

This study follows the Transparent Reporting of a multivariable prediction model for Individual Prognosis or Diagnosis (TRIPOD) report (16). The clinical data of the study were from 2,136 patients with CHF in the Outpatient or Cardiac Rehabilitation Department in Shanghai Tongji Hospital Affiliated to Tongji University from March 1, 2007 to December 31, 2018. The available information includes demographics, medical history, CPET indicators, echocardiography, laboratory testing, and drugs. Other data extracted include the number of hospital admissions, length of stay, and New York Heart Association (NYHA) classification at admission. Inclusion criteria include CHF diagnosed in accordance with the “Chinese Heart Failure Diagnosis and Treatment Guidelines 2018” with a medical history of more than 3 months; age ≥18 years; NYHA heart function level 1–3; and ability to cooperate to complete the CPET. Exclusion criteria include chronic obstructive pulmonary disease, pulmonary heart disease or pulmonary vascular disease; acute myocardial infarction; second or third degree atrioventricular block; acute pericarditis, rheumatic heart disease; thrombophlebitis or intracardiac thrombosis; severe supraventricular or ventricular arrhythmia; and uncontrolled hypertension. According to the criteria of acceptance, 1,589 patients were excluded, and 547 patients with HF were finally included (Figure 1). The study was conducted in accordance with the Declaration of Helsinki as revised in 2013 and was approved by the Ethics Committee of Tongji Hospital Affiliated to Tongji University [LL(H)-08-04]. Informed consent was taken from all the patients. Before analysis, the patient's records/information were anonymized and de-identified.

### Potential Predictive Variables

Potential predictors include the following characteristics of the patient: demographic characteristics (e.g., gender, age, height, weight, and body mass index), basic heart disease history (e.g., coronary heart disease, dilated heart disease, and hypertension), other related medical history (e.g., diabetes, hyperlipidemia, and stroke), CPET indicators [e.g., peak VO_2_, end-tidal CO_2_ pressure (PETCO_2_)], cardiac ultrasonography [e.g., left ventricular ejection fraction (LVEF)], laboratory tests (e.g., blood lipids, and blood potassium), and medications for HF treatment [e.g., angiotensin-converting enzyme inhibitors (ACEI), angiotensin receptor blockers (ARB), angiotensin receptor neprilysin inhibitors (ARNI), β-blockers].

### Study Design

A total of 90 variables were included in the study as potential prognostic factors. Patients with readmission or death records mainly for HF during the follow- up were defined as the readmitted or died group. Patients who did not have a re-admission or death record during the follow up were defined as the not readmitted, alive group. This is an open-label design trial. In order to reduce bias, examiners, researchers collecting data on outcome indicators, data managers, and statisticians do not know the patient's name.

### Outcomes

Readers should refer to the definitions of key clinical data elements and cardiovascular endpoint events issued by the ACC/American Heart Association (AHA) in conjunction with the US Food and Drug Administration and the Cardiovascular Trial Standard Data Collection Program. The endpoint of this study was the composite endpoint of all-cause death and all-cause admission. The data from the electronic medical records and our follow-up results were used to measure this result.

### Follow-Up

Starting from December 2018, a telephone follow-up or electronic medical record review was conducted every 6 months to collect information on hospitalization or death in the past period, until the patient's first readmission or death or the study was terminated by the follow-up on June 30, 2020. The time of hospitalization or death was recorded by the follow-up staff. A wrong telephone number, lack of response, and refusal to follow up were considered as being lost to follow up.

### Statistical Analysis

We used R language [R software (version 3.6.1, R Foundation for Statistical Computing, Vienna, Austria)] and SPSS software (version 20.0; IBM Corp, Armonk, NY, USA) for statistical analysis. The measurement data were tested for normal distribution. Normally distributed measurement data were expressed as mean ± standard deviation. The comparison between two groups was done using an independent sample *t*-test and the comparison between multiple groups using one-way analysis of variance (ANOVA). The least significant difference test was used for multiple comparisons between groups. Non-normal distribution measurement data were expressed as medians and interquartile ranges (IQR) and compared by the non-parametric Mann-Whitney *U*-test. Categorical data were expressed as frequency (percentage), and χ^2^-test was used for comparison between groups. In addition, variables with missing values <20% were subjected to multiple imputation using the R language mice package. *P* < 0.05 indicates that the difference is statistically significant.

### Modeling and Validation

Through random sampling, the entire data set was divided into derivation and validation cohorts in a ratio of 7:3. Least absolute shrinkage and selection operator (LASSO) regression and Cox multivariate analysis were used to screen independent risk factors that could affect the prognosis of CHF, and a nomogram was used to establish a risk prediction model. The C-index values were calculated for the derivation and validation groups. The degree of discrimination was determined and a calibration curve drawn to evaluate the degree of calibration. Finally, the patients were classified into high (greater than the mean risk score) and low risk (no greater than the mean risk score) categories according to the score, and the Kaplan-Meier (KM) curve was drawn for survival analysis (17).

## Results

### Study Population and Cohort Characteristics

A total of 547 patients with HF were screened; 18.3% were women; the median age was 63 years; the median follow-up time was 519 days and the loss to follow up rate was 10.79%; The incidence of composite endpoints (all-cause death and all-cause admission) in 1/3/5/10 years were 40.95%, 56.86%, 60.15%, and 63.44%, respectively. Compared with the not readmitted and alive group, the readmitted or died group had the following characteristics: (i) older subjects; (ii) a high proportion of patients with a history of ischemic heart disease (IHD) (65%), atrial fibrillation (AF) (17.4%), myocardial infarction (MI) (45%), percutaneous coronary intervention (PCI) (48.4%), and diabetes mellitus (DM) (33.6%) (*P* < 0.05); (iii) lower oxygen consumption at anaerobic threshold (VO_2_ AT), peak VO_2_, heart rate at anaerobic threshold (HRAT), and peak average blood pressure (Peak MBP); (iv) higher ventilation/carbon dioxide production (VE/VCO_2_) slope; (v) lower levels of biochemical indicator, troponin I (TnI) (Table 1).

**Table 1:** The characteristics of death or readmission stratification in patients with CHF.

**Characteristic**	**Overall**	**Not readmitted, alive**	**Readmitted or died**	**χ^**2**^/*Z***	***p***
	**(*n* = 547)**	**(*n* = 196)**	**(*n* = 351)**		
Time (median [IQR])	519.00 [193.50, 1444.50]	1609.50 [752.75, 2644.25]	262.00 [127.50, 632.50]	**−14.470**	**0.000**
Female, *n* (%)	100 (18.3)	43 (21.9)	57 (16.2)	2.735	0.098
Age (median [IQR])	63.00 [56.00, 69.00]	60.50 [55.00, 68.00]	64.00 [57.00, 70.00]	**−3.021**	**0.003**
Smoke, *n* (%)	329 (60.1)	119 (60.7)	210 (59.8)	0.041	0.839
Alcohol, *n* (%)	91 (16.6)	32 (16.3)	59 (16.8)	0.021	0.884
IHD, *n* (%)	333 (60.9)	105 (53.6)	228 (65.0)	**6.846**	**0.009**
DCM, *n* (%)	171 (31.3)	71 (36.2)	100 (28.5)	3.501	0.061
AF, *n* (%)	81 (14.8)	20 (10.2)	61 (17.4)	**5.132**	**0.023**
MI, *n* (%)	205 (37.5)	47 (24.0)	158 (45.0)	**23.749**	**0.000**
PCI, *n* (%)	225 (41.1)	55 (28.1)	170 (48.4)	**21.556**	**0.000**
HBP, *n* (%)	386 (70.6)	146 (74.5)	240 (68.4)	2.263	0.132
DM, *n* (%)	158 (28.9)	40 (20.4)	118 (33.6)	**10.684**	**0.001**
Height (median [IQR])	168.31 [166.00, 172.00]	168.31 [165.00, 172.00]	168.31 [166.50, 172.00]	−0.005	0.996
WT (median [IQR])	71.97 [65.00, 76.25]	71.97 [65.00, 80.00]	71.97 [65.00, 76.00]	−0.066	0.947
VO_2_ AT (median [IQR])	10.72 [9.10, 12.20]	10.80 [9.50, 12.83]	10.70 [9.00, 12.00]	**−2.525**	**0.012**
Peak VO_2_ (median [IQR])	14.66 [12.35, 16.90]	14.80 [12.60, 17.70]	14.40 [12.30, 16.15]	**−2.998**	**0.003**
HRAT (median [IQR])	94.00 [85.00, 100.50]	94.50 [86.00, 106.00]	93.00 [84.00, 99.00]	**−3.146**	**0.002**
HR8min (median [IQR])	81.00 [81.00, 81.00]	81.00 [81.00, 81.00]	81.00 [81.00, 81.00]	−0.905	0.366
Peak DBP (median [IQR])	81.00 [72.00, 90.00]	81.00 [75.00, 90.00]	81.00 [72.00, 90.00]	−1.380	0.168
Peak MBP (median [IQR])	105.00 [93.83, 114.33]	107.00 [96.00, 115.42]	105.00 [93.33, 113.67]	**−2.091**	**0.037**
VE/VCO_2_ (median [IQR])	34.90 [30.65, 39.10]	34.00 [29.26, 38.12]	35.75 [31.50, 40.15]	**−3.088**	**0.002**
LVEF (median [IQR])	0.45 [0.36, 0.52]	0.45 [0.38, 0.50]	0.45 [0.36, 0.52]	−0.040	0.968
LVEF group				3.004	0.223
LVEF <0.4, *n* (%)	170 (31.1)	57 (29.1)	113 (32.2)		
LVEF 0.4–0.49, *n* (%)	222 (40.6)	89 (45.4)	133 (37.9)		
LVEF ≥0.5, *n* (%)	155 (28.3)	50 (25.5)	105 (29.9)		
CRP (median [IQR])	6.40 [3.00, 6.40]	6.40 [3.00, 6.40]	6.40 [3.00, 6.40]	−0.562	0.574
BNP (median [IQR])	702.00 [702.00, 702.00]	702.00 [702.00, 702.00]	702.00 [702.00, 702.00]	−0.750	0.453
TnI (median [IQR])	0.05 [0.01, 0.39]	0.08 [0.01, 0.39]	0.05 [0.01, 0.39]	**−1.988**	**0.047**
UA (median [IQR])	432.00 [355.00, 464.00]	432.00 [368.75, 456.00]	432.00 [353.00, 469.50]	−0.244	0.807

*Significant values of P <0.05 is indicated in bold*.

*AF, atrial fibrillation; BNP, B-type natriuretic peptide; CRP, C-reactive protein; DCM, dilated cardiomyopathy; DM, diabetes mellitus; HBP, high blood pressure; HR8min, heart rate at the 8th minute after the cardiopulmonary exercise peaked; HRAT, heart rate at anaerobic threshold; IHD, ischemic heart disease; LVEF, left ventricular ejection fraction; MI, myocardial infarction; PCI, percutaneous coronary intervention; Peak DBP, peak diastolic blood pressure; Peak MBP, peak average blood pressure; Peak VO_2_, peak oxygen uptake; TnI, troponin I; UA, uric acid; VE/VCO_2_, ventilation/carbon dioxide production; VO_2_AT, oxygen consumption at anaerobic threshold; WT, weight*.

### Risk Model Development and Validation

Using computer-generated random numbers, the majority (70%) of the cohort were randomly assigned to the derivation cohort (*n* = 384), and the remaining 30% were assigned to the validation cohort (*n* = 163). There was no significant difference in the characteristics between the derivation and validation groups (Supplementary Table 1). After 90 variables entered LASSO regression screening, 14 variables [age, AF, MI, PCI, DM, height, peak VO_2_, heart rate at the 8th minute after the cardiopulmonary exercise peaked (HR8min), peak diastolic blood pressure (peak DBP), peak MBP, c-reactive protein (CRP), b-type natriuretic peptide (BNP), TnI, uric acid (UA)] were obtained (Supplementary Figures 1A,B), and eight variables were retained after univariate analysis, multivariable analysis, and stepwise regression (Table 2; Supplementary Table 2).

**Table 2:** Analysis of risk events indicators in chronic heart failure patients.

**Indicators**	**Univariate analysis**		**Multivariable analysis**	
	**HR (95% CI)**	***P***	**HR (95% CI)**	***P***
Age	1.03 (1.02–1.04)	**0.000**	1.03 (1.01–1.04)	**0.000**
AF	1.80 (1.30–2.49)	**0.000**	1.60 (1.12–2.29)	**0.009**
MI	1.73 (1.34–2.24)	**0.000**	1.26 (0.72–2.20)	0.417
PCI	1.70 (1.31–2.20)	**0.000**	1.81 (1.02–3.18)	**0.041**
DM	1.56 (1.18–2.05)	**0.002**	1.41 (1.06–1.88)	**0.020**
Height	1.01 (0.99–1.03)	0.175	1.01 (1.00–1.03)	0.124
Peak VO_2_	0.93 (0.90–0.97)	**0.000**	0.95 (0.91–0.99)	**0.012**
HR8min	1.05 (1.02–1.08)	**0.004**	1.06 (1.03–1.10)	**0.000**
Peak DBP	0.99 (0.98–1.00)	**0.008**	1.00 (0.98–1.02)	0.696
Peak MBP	0.99 (0.98–1.00)	**0.010**	0.99 (0.98–1.01)	0.538
CRP	1.03 (1.01–1.05)	**0.000**	1.04 (1.02–1.05)	**0.000**
BNP	1.00 (1.00–1.00)	**0.003**	1.00 (1.00–1.00)	0.108
TnI	0.93 (0.83–1.05)	0.243	0.92 (0.81–1.04)	0.187
UA	1.00 (1.00–1.00)	**0.016**	1.00 (1.00–1.00)	**0.005**

*Significant values of P < 0.05 is indicated in bold*.

*AF, atrial fibrillation; BNP, b-type natriuretic peptide; CRP, c-reactive protein; DM, diabetes mellitus; HR8min, heart rate at the 8th minute after the cardiopulmonary exercise peaked; MI, myocardial infarction; PCI, percutaneous coronary intervention; Peak DBP, peak diastolic blood pressure; Peak MBP, peak average blood pressure; Peak VO_2_, peak oxygen uptake; TnI, troponin I; UA, uric acid*.

**Figure 2:**
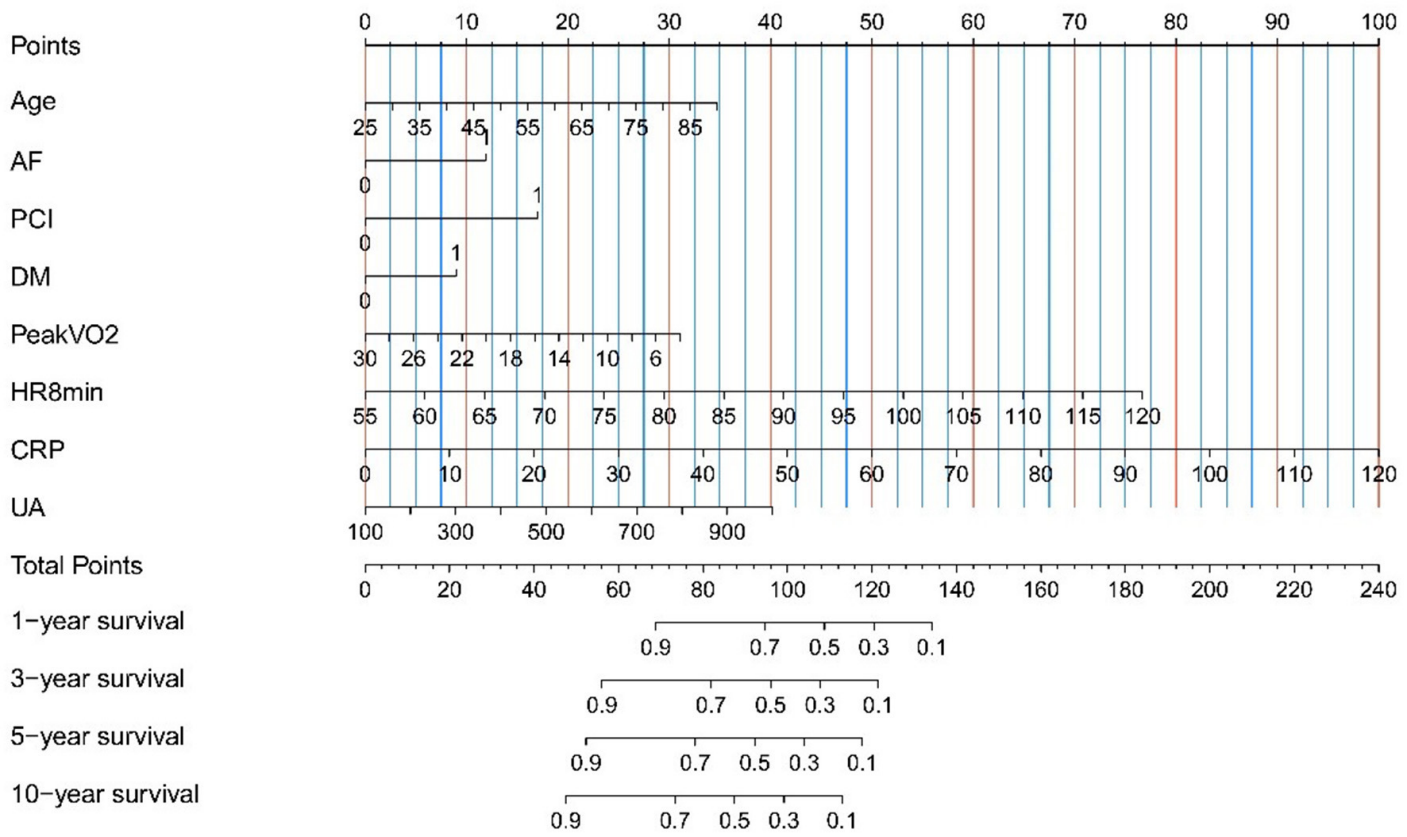
Nomogram for the prediction of mortality and hospitalization in Chinese heart failure patients. Instructions for use of nomogram: draw a vertical line, and then add up the points corresponding to each feature, and then draw the vertical line according to the total points until it intersects each survival axis to determine the survival probability of 1, 3, 5, and 10 years. For binary variables, 0 = no, 1 = yes. AF, atrial fibrillation; CRP, c-reactive protein; DM, diabetes mellitus; HR8min, heart rate at the 8th minute after the cardiopulmonary exercise peaked; PCI, percutaneous coronary intervention; Peak VO_2_, peak oxygen uptake; UA, uric acid.

In univariate regression analysis, 12 variables were significantly related to the prognosis of heart failure (the composite endpoint of death and rehospitalization). In the multivariate Cox regression analysis, the regression coefficients of the indicators in Table 3, and construct the prognostic risk proportion model [the calculation formula is h(t,x) = h0 (t)exp(0.025Age+0.560AF+0.803PCI+0.423DM−0.056Peak VO2+0.056HR8min+0.039 CRP+0.002UA)] according to the multivariate Cox regression coefficients of the eight indicators (18). Factors—age, AF, PCI, DM, peak VO_2_, HR8min, CRP, and UA—that were independently associated with a higher likelihood of readmission or death were construct a risk scoring model, which was expressed in the form of a nomogram (Tables 2, 3; Figure 2; Supplementary Figure 2).

**Table 3:** The selected variables for model construction.

**Factors**	**Coefficient**	**HR (95%CI)**	***P***
Age	0.025	1.03 (1.01–1.04)	0.000
AF	0.560	1.75 (1.24–2.47)	0.001
PCI	0.803	2.23 (1.68–2.96)	0.000
DM	0.423	1.53 (1.16–2.02)	0.003
Peak VO_2_	–0.056	0.95 (0.91–0.98)	0.005
HR8min	0.056	1.06 (1.02–1.09)	0.001
CRP	0.039	1.04 (1.03–1.05)	0.000
UA	0.002	1.00 (1.00–1.00)	0.000

*AF, atrial fibrillation; CRP, c-reactive protein; DM, diabetes mellitus; HR8min, heart rate at the 8th minute after the cardiopulmonary exercise peaked; PCI, percutaneous coronary intervention; Peak VO_2_, peak oxygen uptake; UA, uric acid*.

The AUC of the prediction model is shown in Supplementary Figure 3. To evaluate the effectiveness of risk prediction for the composite endpoint of heart failure death and readmission. The sensitivity, specificity, positive predictive value, and negative predictive value of the training group's prediction model are shown in Supplementary Table 3.

In the derivation and validation cohorts, the calculated model discrimination C-index values were 0.69 (95%CI 0.65–0.72) and 0.62 (95%CI 0.57–0.67), respectively, and the model's discrimination ability was moderate. The calibration chart shows that the occurrence of end-point events at 1 year/3 years/5 years/10 years was in good agreement with the actual observations (Supplementary Figure 4).

**Figure 3:**
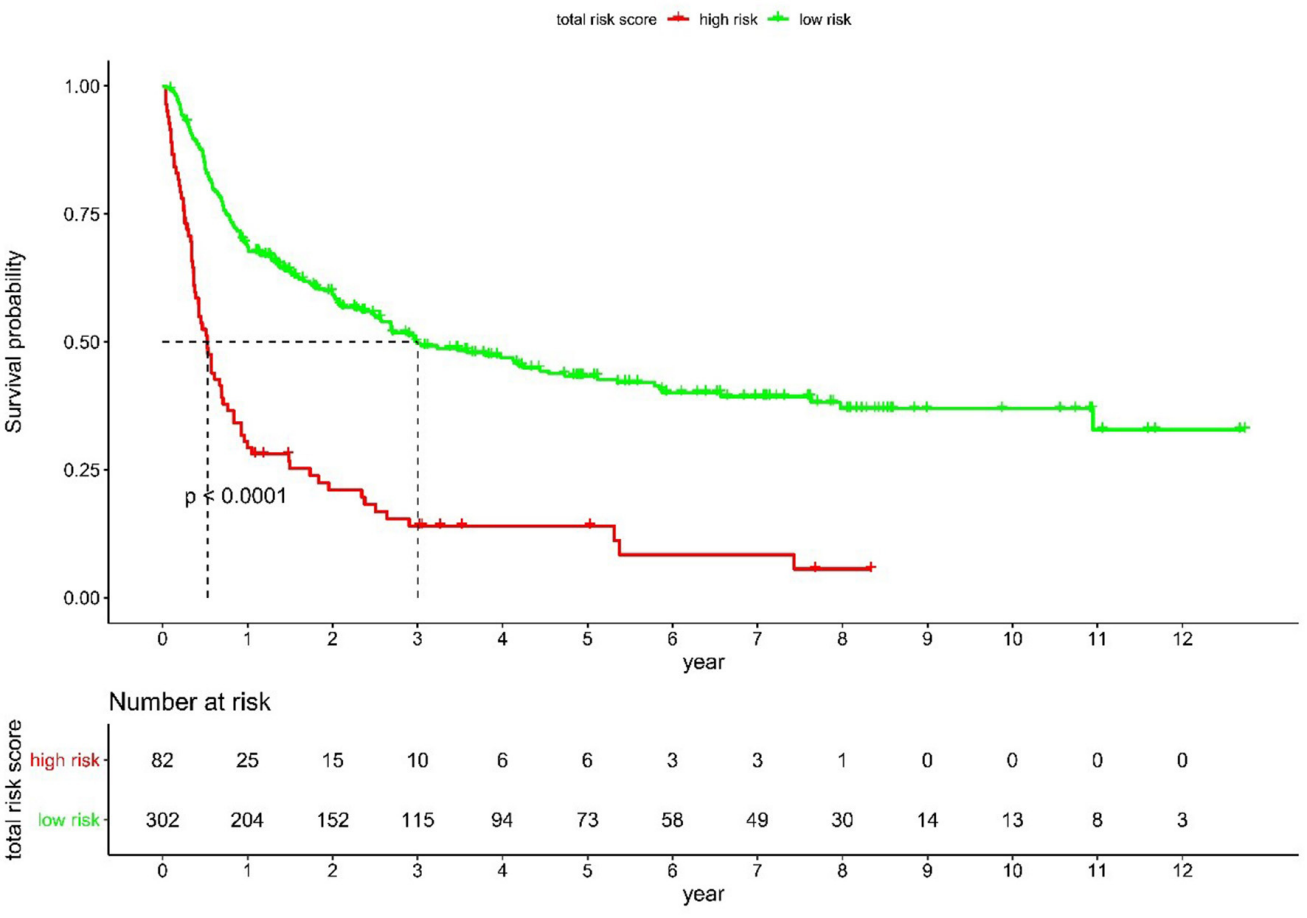
Kaplan–Meier cumulative event rates in the derivation cohort.

Based on the score calculated by the nomogram, the derivation cohort was divided into two groups, high- and low- risk groups, and KM curve survival analysis was performed. The results of the LogRank method showed that the KM curves of the high-risk and low-risk groups were significantly different in the three cohorts (derivation group, validation group, and the whole group). In the derivation group, the risk of rehospitalization or death at 1 year/3 years/5 years/10 years was 69.5, 87.8, 92.7, and 100% for the high-risk group and 32.5,61.9, 75.8, and 95.7% for the low-risk group, respectively (Figure 3; Supplementary Figure 5).

## Discussion

In this study, we selected simple parameters based on clinical conditions, laboratory indicators, cardiopulmonary exercise tests, and other measurements, and developed a new prediction model based on the Chinese HF population, which can assess the risk of readmission or death of patients with CHF. The C index values of the derivation cohort and validation cohorts were 0.69 (95%CI 0.65–0.72) and 0.62 (95%CI 0.57–0.67), respectively, which indicate moderate levels of predictive ability. Our C-index values are high among those from previously published HF risk prediction models (19). Moreover, the calibration curve at 1 year/3 years/5 years/10 years showed good consistency. Compared with the traditional HF risk prediction model, our model has some advantages for CHF patients in China: (i) it adds relevant CPET indicators; (ii) has a long follow-up time, and (iii) the target population is based on the Chinese population. This model can comprehensively assess the long-term prognosis of patients with HF and provide data for the Chinese population for inclusion in the world's CHF prognosis assessment.

In our cohort study, it was observed that patients with CHF who were hospitalized or who died were older, had more complications, and had poorer exercise endurance. The indicators included in the model were age, AF, PCI, DM, peak VO_2_, HR8min, CRP, and UA. As a protective factor, peak VO_2_ has been used as a tool to assess the severity of the disease, judge the short-term and long-term prognosis of patients with HF, and select patients for heart transplantation (20, 21). Lewis and Zlotoff (22) summarized the application of risk stratification based on cardiopulmonary exercise experiments in the management of advanced HF. They showed that exercise response patterns are predictive: Peak oxygen consumption can predict the lifespan of ordinary people, and CPET can effectively divide patients into high-risk and low-risk categories of HF events (22). Of note, as the peak VO_2_ index can be improved by exercise (23), this model evaluation encourages high-risk HF patients to undergo exercise-based cardiac rehabilitation. Therefore, we believe that during continuous exercise, with the improvement of exercise endurance, the risk score may continue to improve, and finally improve the long-term prognosis.

In addition, we noted that the risk factors that affect the prognosis also include heart rate (specific time), CRP, and UA, which is consistent with the risk factors mentioned in the current study (24–30). Heart rate is a determinant of myocardial oxygen demand, coronary blood flow, and myocardial function, and is the key to adapting cardiac output to metabolic demands. An elevated heart rate can predict adverse outcomes in patients with CHF, which may be related to neurohormonal activation (24). Inflammation plays a key role in the etiology and progression of atherosclerosis. Studies have shown that in patients undergoing PCI for the first ST elevation myocardial infarction, there is a clear relationship between the in-hospital CRP plasma concentration and the development of HF after infarction (25). Uric acid can affect cardiovascular disease through platelet aggregation and endothelial inflammation activation. The change in serum uric acid levels in the hospital can predict adverse outcomes in patients with HF (26, 31).

There are other combined CPET HF risk scores, whose predictive accuracy is variable, depending on population selection, treatment, event selection, and follow-up time (32–35). The 2012 Heart Failure: A Controlled Trial Investigating Outcomes of Exercise TraiNing (HF-ACTION) predictive risk score model is the first risk predictive model for HF patients caused by systolic insufficiency. It analyzes multiple candidate variables, including demographics, medical history, laboratory values, CPET exercise parameters, quality of life, and the level of depression (32). The HF-ACTION represents heart failure with reduced ejection fraction (HFrEF) patients, and its score contains indicators that are easily available. However, the limitation is that patients with preserved systolic function are excluded, and the representativeness of people from outside the United States are limited. Our study included patients with heart failure with a moderately reduced ejection fraction (HFmrEF) and heart failure with preserved ejection fraction (HFpEF), and included the Chinese population for modeling, which has good applicability to the Chinese population. At the same time, the indicators we included are easily available clinically. In 2013, the Metabolic Exercise test data combined with Cardiac and Kidney Indexes (MECKI) score combined CPET indicators with established clinical, laboratory and echocardiography parameters, and finally included six indicators of hemoglobin, Na+, modification of diet in renal disease, LVEF, peak VO_2_, and VE/VCO_2_ slope (33). These continuous variables define the MECKI score, identify the risk of cardiovascular death and heart transplantation, and have been validated in different settings (34). However, it is mainly used for white people, and its applicability to the Chinese population is unknown. In 2020, Pugliese et al. identified five independent predictors including peak oxygen consumption, which can determine the increased risk of adverse events in patients with HF with preserved ejection fraction (35). However, the sample size of that study is small, the follow-up time is short, and its applicability in Chinese population needs to be investigated.

Although there are many HF risk prediction models in the world, a model based on Chinese population data is an unmet need. On the one hand, our current model highlights the importance of CPET in risk scoring, helps optimize the risk stratification and management of HF patients, and supports cardiac rehabilitation programs. On the other hand, the indicators included in our model are simple and easy to obtain, which helps its vigorous promotion in primary hospitals.

Our study has the following limitations: First, as all the patients are from Shanghai Tongji Hospital, this is a single-center study with regional limitations and an insufficient sample size. Our future research will include people from other parts of China and expand the sample size of the research. Second, the included population comprises people who are around 60 years old, which may limit the generality of our model. However, compared with younger patients, older patients tend to have a higher risk of death and readmission. Third, there is currently a lack of external verification. At the same time, there is also a lack of comparison data with other international forecasting models in the same Chinese cohort. In the future, we will promote this model, conduct external verification, compare it with other international models in the same Chinese cohort, and continue to improve the model to provide a theoretical basis for treatment decisions for Chinese HF patients. Finally, the loss to follow-up rate in this study exceeded 5%. The main reasons include patients' lack of attention to the study, poor compliance, and insufficient communication skills of the follow-up staff. Moreover, due to the long follow-up time (the longest follow-up time is up to 12 years), the patient's telephone number and place of residence have changed, resulting in a high rate of loss to follow-up. In the future follow-up, we will strengthen patient education, increase doctor-patient contact, and conduct communication training for follow-up personnel, so as to reduce the rate of loss to follow-up as much as possible.

## Conclusions

In this study, a scoring model for CHF in China was constructed based on CPET indicators. This model can evaluate the long-term risk of death or rehospitalization due to CHF and provide decision-making basis for clinicians, patients, and their families.

### Clinical Perspectives

Clinicians use this model to assess the prognosis of patients with heart failure and identify high-risk patients, so as to better guide the implementation of treatment plans.

### Translational Outlook

Although there are many models for evaluating the mortality and hospitalization risk of patients with heart failure, this study combines the experimental indicators of cardiopulmonary exercise to propose a long-term prognosis and readmission model for chronic heart failure in the Chinese population, laying the foundation for more accurate risk stratification in the future.

## Data Availability Statement

The raw data supporting the conclusions of this article will be made available by the authors, without undue reservation.

## Ethics Statement

The studies involving human participants were reviewed and approved by Ethics Committee of Tongji Hospital Affiliated to Tongji University. The patients/participants provided their written informed consent to participate in this study.

## Author Contributions

BZ and TS prepared the manuscript and all the authors participated in the clinical and related research. All authors gave final approval and agreed to be accountable for the integrity and accuracy of all aspects of the work.

## Funding

This work was supported by National Natural Science Foundation (81974359 and 81700316) and advanced proper technology promotion project of Municipal Health and Health Commission in Shanghai, China (2019SY014).

## Conflict of Interest

The authors declare that the research was conducted in the absence of any commercial or financial relationships that could be construed as a potential conflict of interest.

## Publisher's Note

All claims expressed in this article are solely those of the authors and do not necessarily represent those of their affiliated organizations, or those of the publisher, the editors and the reviewers. Any product that may be evaluated in this article, or claim that may be made by its manufacturer, is not guaranteed or endorsed by the publisher.

## Abbreviations

ACC: American College of Cardiology
ACEI: angiotensin-converting enzyme inhibitors
AF: atrial fibrillation
AHA: American Heart Association
ANOVA: analysis of variance
ARB: angiotensin receptor blockers
ARNI: angiotensin receptor neprilysin inhibitor
BIOSTAT-CHF: Biology Study to Tailored Treatment in Chronic Heart Failure
BNP: B-type natriuretic peptide
CHF: chronic heart failure
CPETs: cardiopulmonary exercise tests
CRP: C-reactive protein
DCM: dilated cardiomyopathy
DM: diabetes mellitus
HBP: high blood pressure
HF: heart failure
HF-ACTION: Heart Failure: A Controlled Trial Investigating Outcomes of Exercise TraiNing
HFmrEF: heart failure with a moderately reduced ejection fraction
HFpEF: heart failure with preserved ejection fraction
HFrEF: heart failure with reduced ejection fraction
HR8min: heart rate at the 8th minute after the cardiopulmonary exercise peaked
HRAT: heart rate at anaerobic threshold
IHD: ischemic heart disease
IQR: interquartile ranges
KM: Kaplan-Meier
LASSO: least absolute shrinkage and selection operator
LVEF: left ventricular ejection fraction
MECKI: The Metabolic Exercise test data combined with Cardiac and Kidney Indexes
MI: myocardial infarction
NYHA: New York Heart Association
PARADIGM-HF: Global Mortality and Morbidity in Heart Failure
PCI: percutaneous coronary intervention
Peak DBP: peak diastolic blood pressure
Peak MBP: peak average blood pressure
Peak VO_2_: peak oxygen uptake
PETCO_2_: end-tidal CO_2_ pressure
TnI: troponin I
TRIPOD: transparent reporting of a multivariable prediction model for individual prognosis or diagnosis
UA: uric acid
VE/VCO_2_: ventilation/carbon dioxide production
VO_2_ AT: oxygen consumption at anaerobic threshold
WT: weight.
