# Supplementary material for: A Model for the Prediction of Mortality and Hospitalization in Chinese Heart Failure Patients
Source: Front Cardiovasc Med. 2021 Nov 18;8:761605. doi: 10.3389/fcvm.2021.761605 (PMC8639158; doi:10.3389/fcvm.2021.761605)
Supplement: Supplementary file 1 [file Data_Sheet_1.docx]

Supplementary online content

eTable 1

| Characteristic | Full cohort  (n=547) | Derivation group  (n=384) | Validation group  (n=163) | χ2/Z | p |
| --- | --- | --- | --- | --- | --- |
| Time (median [IQR]) | 519.00[193.50, 1444.50] | 544.50 [195.50, 1529.50] | 441.00[193.00, 1089.00] | -1.276 | 0.202 |
| Female, n (%) | 100 (18.3) | 70 (18.2) | 30 (18.4) | 0.002 | 0.961 |
| Age (median [IQR]) | 63.00 [56.00, 69.00] | 63.00 [56.00, 69.00] | 63.00 [56.00, 70.00] | -0.315 | 0.753 |
| Smoke, n (%) | 329(60.1) | 226(58.9) | 103(63.2) | 0.898 | 0.343 |
| Alcohol, n (%) | 91(16.6) | 66(17.2) | 25(15.3) | 0.282 | 0.595 |
| IHD, n (%) | 333 (60.9) | 230 (59.9) | 103 (63.2) | 0.521 | 0.470 |
| DCM, n (%) | 171 (31.3) | 124 (32.3) | 47 (28.8) | 0.637 | 0.425 |
| AF, n (%) | 81 (14.8) | 59 (15.4) | 22 (13.5) | 0.316 | 0.574 |
| MI, n (%) | 205 (37.5) | 142 (37.0) | 63 (38.7) | 0.136 | 0.712 |
| PCI, n (%) | 225 (41.1) | 155 (40.4) | 70 (42.9) | 0.315 | 0.575 |
| HBP, n (%) | 386 (70.6) | 268 (69.8) | 118 (72.4) | 0.373 | 0.542 |
| DM , n (%) | 158 (28.9) | 108 (28.1) | 50 (30.7) | 0.362 | 0.547 |
| Height (median [IQR]) | 168.31 [166.00, 172.00] | 168.31 [165.00, 172.00] | 168.31 [167.50, 173.00] | -1.345 | 0.179 |
| WT (median [IQR]) | 71.97 [65.00, 76.25] | 71.97 [65.00, 76.00] | 71.97 [66.50, 78.00] | -0.813 | 0.416 |
| VO2 AT (median [IQR]) | 10.72 [9.10, 12.20] | 10.72 [9.00, 12.30] | 10.72 [9.50, 12.00] | -0.072 | 0.943 |
| PeakVO2 (median [IQR]) | 14.66 [12.35, 16.90] | 14.66 [12.30, 16.90] | 14.66 [12.75, 16.75] | -0.262 | 0.793 |
| HRAT (median [IQR]) | 94.00 [85.00, 100.50] | 94.00 [86.00, 101.00] | 94.00 [84.00, 100.00] | -0.396 | 0.692 |
| HR8min (median [IQR]) | 81.00 [81.00, 81.00] | 81.00 [81.00, 81.00] | 81.00 [81.00, 81.00] | -0.066 | 0.947 |
| Peak DBP (median [IQR]) | 81.00 [72.00, 90.00] | 81.00 [72.00, 90.00] | 81.00 [74.00, 90.00] | -0.573 | 0.567 |
| Peak MBP (median [IQR]) | 105.00 [93.83, 114.33] | 105.00 [94.83, 113.67] | 105.00 [93.33, 115.50] | -0.570 | 0.569 |
| VE/VCO2 (median [IQR]) | 34.90 [30.65, 39.10] | 35.30 [30.80, 40.00] | 34.00 [30.05, 37.20] | -2.525 | 0.012 |
| LVEF (median [IQR]) | 0.45 [0.36, 0.52] | 0.45 [0.37, 0.50] | 0.45 [0.36, 0.55] | -0.369 | 0.712 |
| LVEF group |  |  |  | 2.627 | 0.269 |
| LVEF<0.4, n (%) | 170 (31.1) | 117 (30.5) | 53 (32.5) |  |  |
| LVEF 0.4-0.49, n (%) | 222 (40.6) | 164 (42.7) | 58 (35.6) |  |  |
| LVEF ≥0.5, n (%) | 155 (28.3) | 103 (26.8) | 52 (31.9) |  |  |
| CRP (median [IQR]) | 6.40 [3.00, 6.40] | 6.40 [3.00, 6.40] | 6.40 [2.57, 6.40] | -0.422 | 0.673 |
| BNP (median [IQR]) | 702.00 [702.00, 702.00] | 702.00 [702.00, 702.00] | 702.00 [702.00, 702.00] | -1.177 | 0.239 |
| TnI (median [IQR]) | 0.05 [0.01, 0.39] | 0.05 [0.01, 0.39] | 0.05 [0.01, 0.39] | -0.142 | 0.887 |
| UA (median [IQR]) | 432.00 [355.00, 464.00] | 432.00 [359.00, 472.50] | 432.00 [352.00, 434.50] | -1.499 | 0.134 |

Abbreviations: AF, atrial fibrillation; BNP, B-type natriuretic peptide; CRP, C-reactive protein ; DCM, dilated cardiomyopathy; DM, diabetes mellitus; HBP, high blood pressure ; HR8min, heart rate at the 8th minute after the cardiopulmonary exercise peaked; HRAT, heart rate at anaerobic threshold ; IHD, ischemic heart disease; LVEF, left ventricular ejection fraction ; MI, myocardial infarction ; PCI, percutaneous coronary intervention; Peak DBP, peak diastolic blood pressure; Peak MBP, peak average blood pressure; Peak VO2, peak oxygen uptake; TnI, troponin I; UA, uric acid; VE/VCO2, ventilation/carbon dioxide production ; VO2AT, oxygen consumption at anaerobic threshold; WT, weight.

eTable2 Stepwise regression

eTable2A Forward Stepwise (Likelihood Ratio)

| Step 9 |  | B | SE | Wald | df | Sig. | Exp(B) |
| --- | --- | --- | --- | --- | --- | --- | --- |
|  | Age | .018 | .005 | 12.477 | 1 | .000 | 1.018 |
|  | AF | .333 | .146 | 5.216 | 1 | .022 | 1.396 |
|  | PCI | .663 | .114 | 33.569 | 1 | .000 | 1.941 |
|  | DM | .272 | .117 | 5.443 | 1 | .020 | 1.313 |
|  | PeakVO2 | -.042 | .016 | 7.196 | 1 | .007 | .959 |
|  | CRP | .020 | .006 | 10.990 | 1 | .001 | 1.021 |
|  | UA | .001 | .000 | 6.106 | 1 | .013 | 1.001 |

Abbreviations: AF, atrial fibrillation; CRP, c-reactive protein; DM, diabetes mellitus; PCI, percutaneous coronary intervention ; Peak VO2, peak oxygen uptake; UA, uric acid.

eTable2B Backward Stepwise (Likelihood Ratio)

| Step 5 |  | B | SE | Wald | df | Sig. | Exp(B) |
| --- | --- | --- | --- | --- | --- | --- | --- |
|  | Age | .017 | .005 | 10.760 | 1 | .001 | 1.017 |
|  | AF | .298 | .147 | 4.089 | 1 | .043 | 1.347 |
|  | MI | .673 | .114 | 34.589 | 1 | .000 | 1.960 |
|  | DM | .261 | .118 | 4.875 | 1 | .027 | 1.299 |
|  | PeakVO2 | -.033 | .015 | 4.562 | 1 | .033 | .968 |
|  | HR8min | .024 | .012 | 3.915 | 1 | .048 | 1.024 |
|  | PeakDBP | -.009 | .004 | 4.996 | 1 | .025 | .991 |
|  | CRP | .020 | .006 | 9.815 | 1 | .002 | 1.020 |
|  | BNP | .000 | .000 | 4.000 | 1 | .046 | 1.000 |
|  | UA | .001 | .000 | 3.571 | 1 | .059 | 1.001 |

Abbreviations: AF, atrial fibrillation; BNP, b-type natriuretic peptide; CRP, c-reactive protein ;DM, Diabetes mellitus; HR8min, heart rate at the 8th minute after the cardiopulmonary exercise peaked; MI, myocardial infarction ; Peak DBP, peak diastolic blood pressure; Peak VO2, peak oxygen uptake; UA, uric acid.

eTable3

Evaluation of this model in the risk prediction of mortality and hospitalization in heart failure patients

|  | Sen | Spe | PPV | NPV |
| --- | --- | --- | --- | --- |
| Derivation |  |  |  |  |
| 1-year | 0.35 | 0.91 | 0.72 | 0.68 |
| 3-year | 0.50 | 0.84 | 0.81 | 0.55 |
| 5-year | 0.96 | 0.14 | 0.66 | 0.67 |
| 10-year | 0.53 | 0.92 | 0.94 | 0.46 |
| Validation |  |  |  |  |
| 1-year | 0.99 | 0.03 | 0.45 | 0.75 |
| 3-year | 0.24 | 0.98 | 0.95 | 0.42 |
| 5-year | 0.22 | 0.97 | 0.94 | 0.37 |
| 10-year | 0.09 | 0.99 | 0.99 | 0.18 |

Abbreviations: Sen, sensitivity; Spe, specificity; PPV, positive predictive value; NPV, negative predictive value.

eFigure1 Feature selection using the least absolute shrinkage and selection operator (LASSO)binary logistic regression model. (A) LASSO coefficient profiles of the 90 baseline features. (B) Tuning parameter(λ)selection in the LASSO model used 10-fold cross-validation via minimum criteria. eFigure2. Multivariate cox regression analysis

eFigure3. The area under the receiver-operator characteristic(ROC) curve(AUC) of predicting readmission or death among patients with heart failure.(A)(B)(C)

eFigure4. Derivation and validation group calibration curve.

eFigure5. Validation and full cohort survival curve


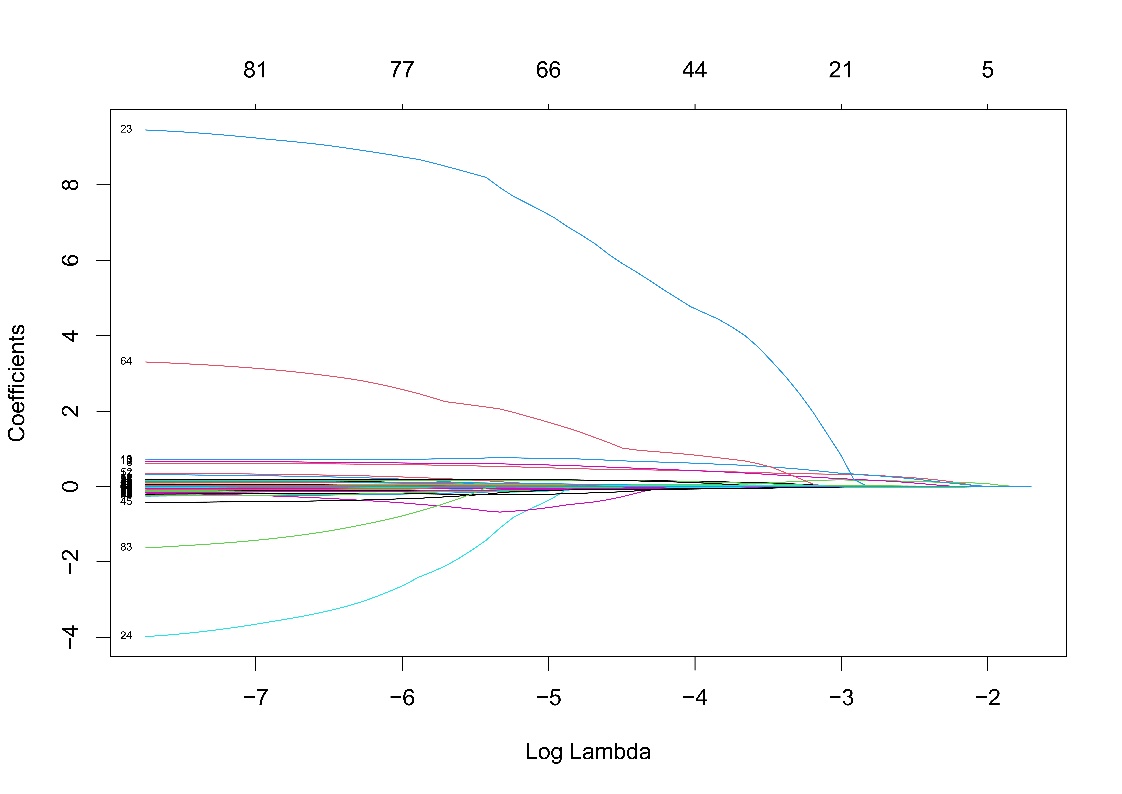


eFigure1 A


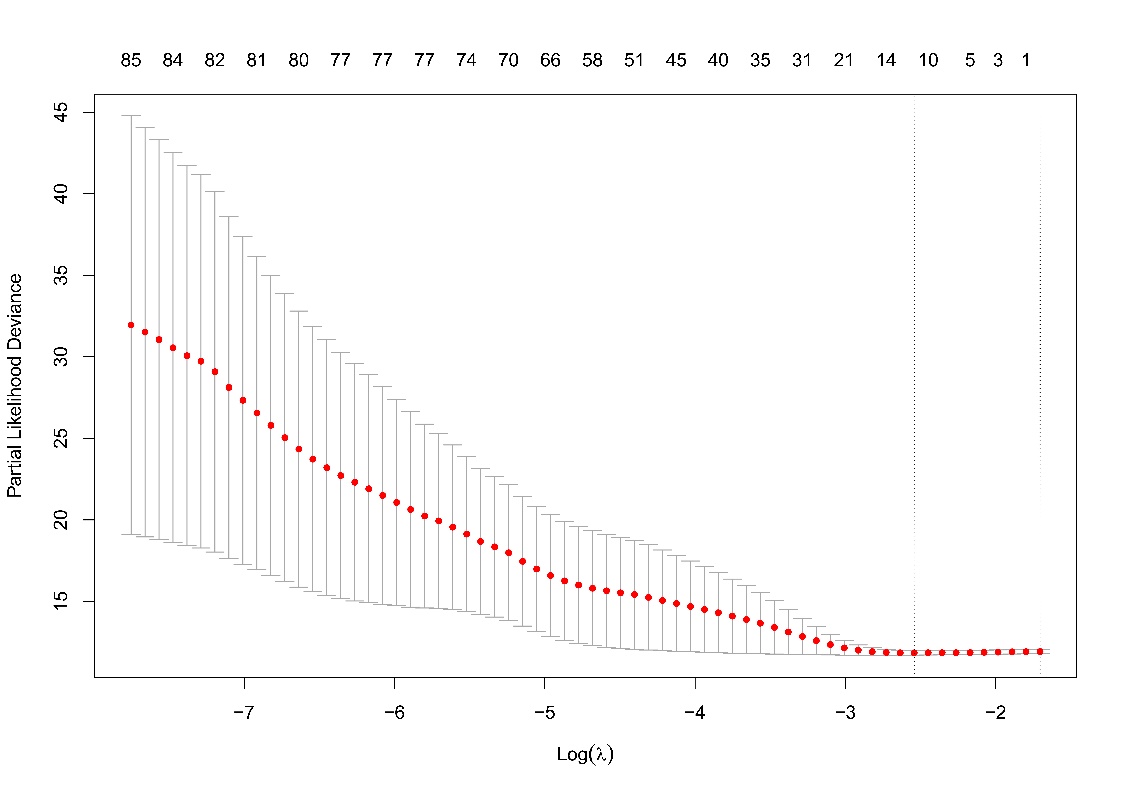


eFigure1 B

eFigure 1 Feature selection using the least absolute shrinkage and selection operator (LASSO)binary logistic regression model. (A) LASSO coefficient profiles of the 90 baseline features. (B) Tuning parameter(λ)selection in the LASSO model used 10-fold cross-validation via minimum criteria.


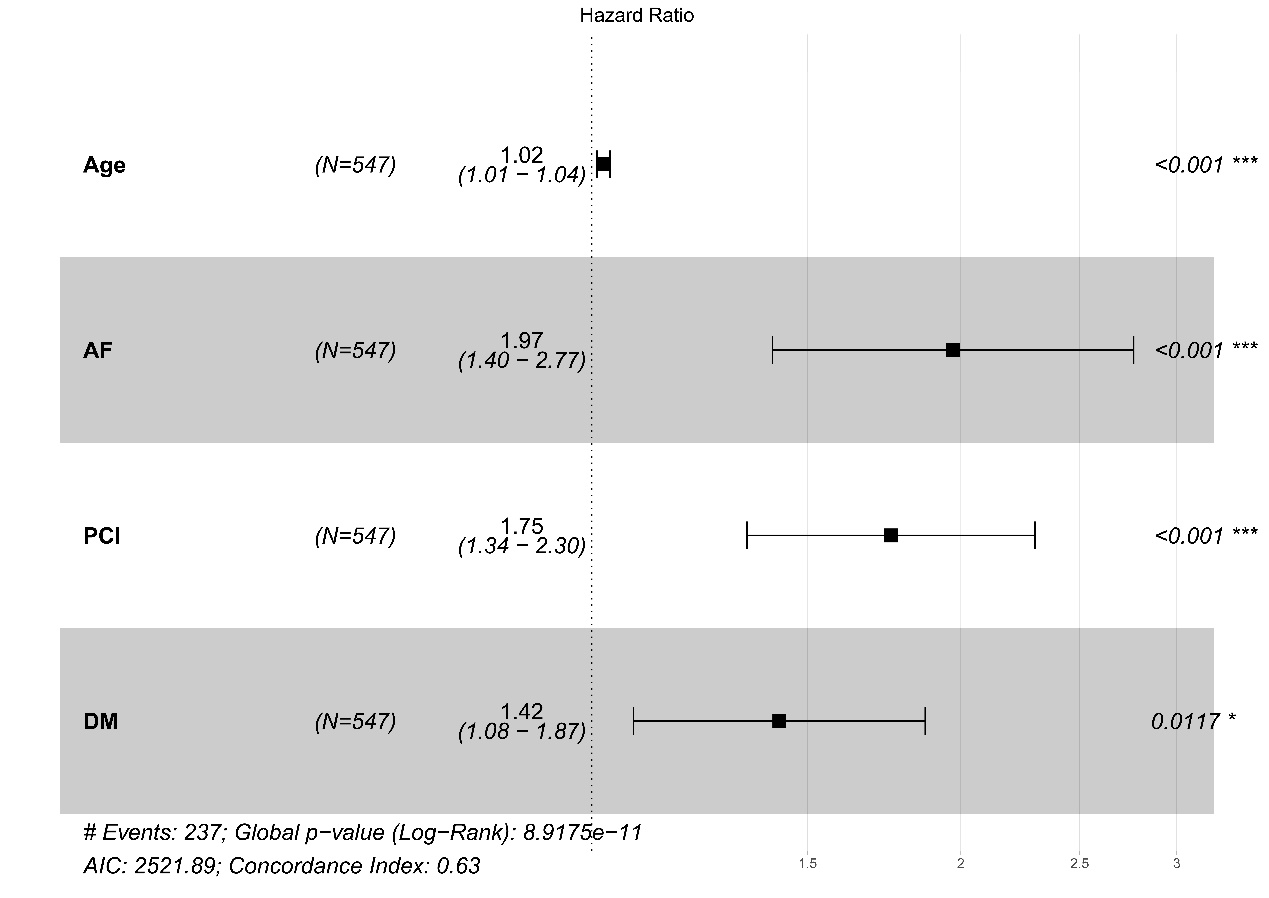


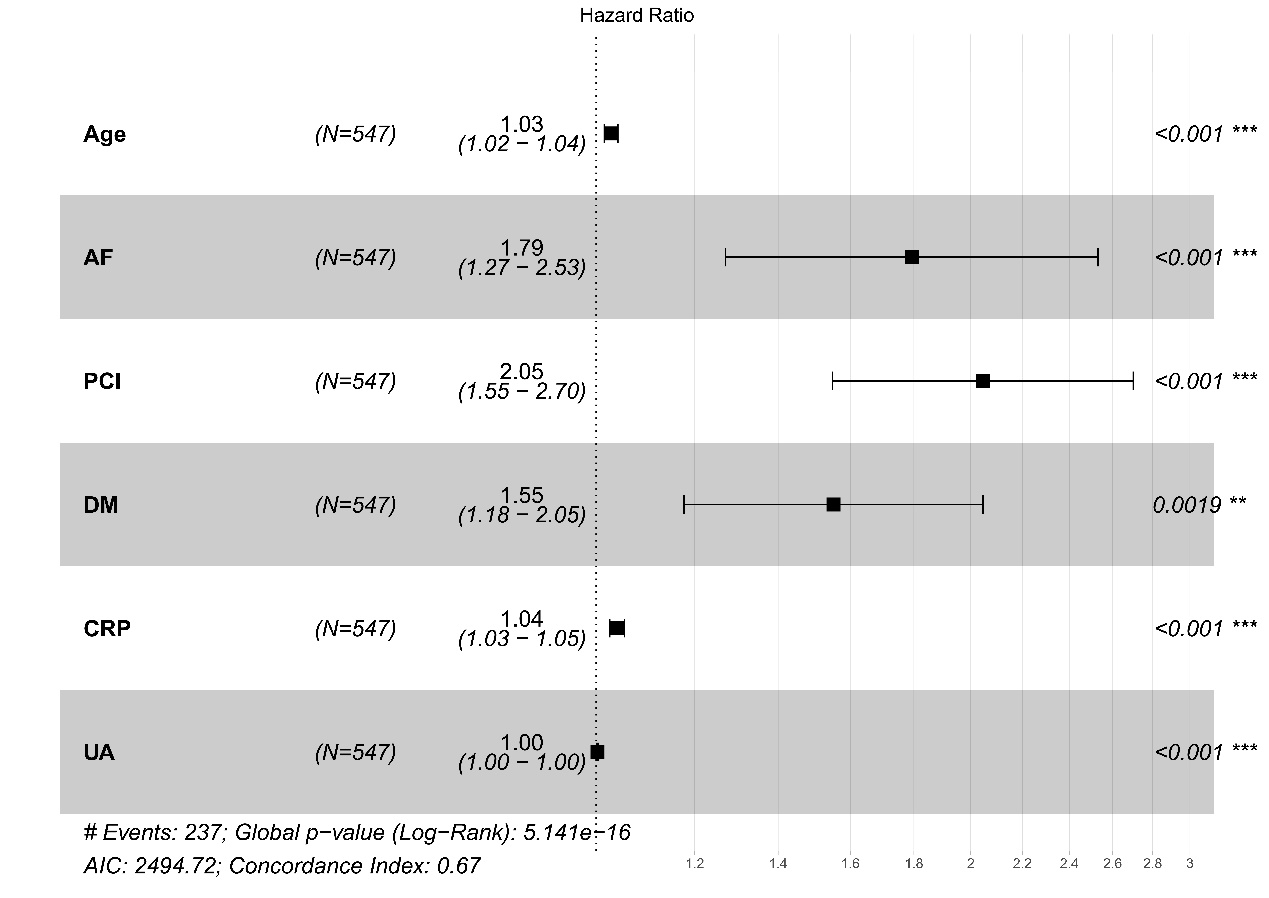


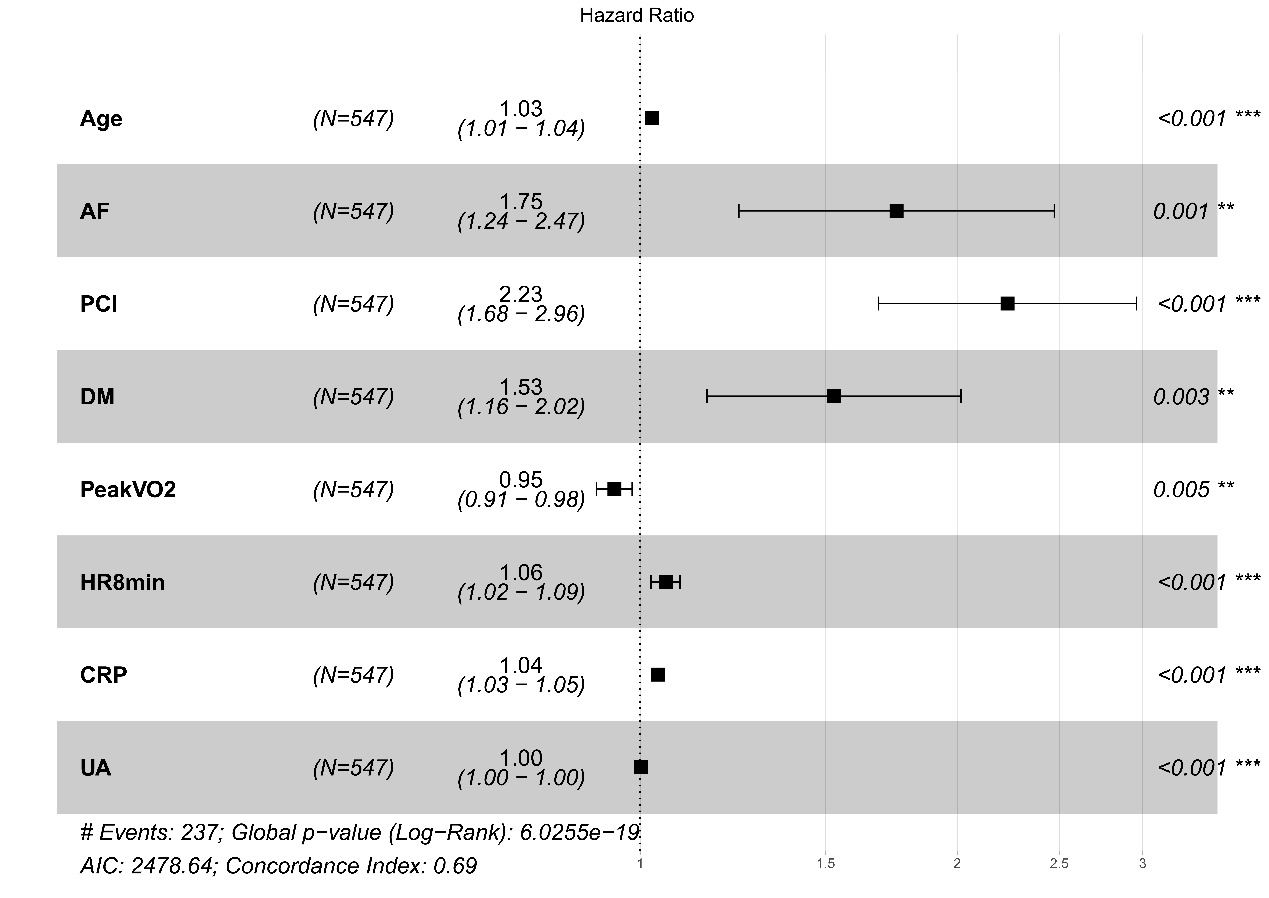


eFigure2. Multivariate cox regression analysis

Abbreviations: AF, atrial fibrillation; CRP, c-reactive protein; DM, diabetes mellitus; HR8min, heart rate at the 8th minute after the cardiopulmonary exercise peaked; PCI, percutaneous coronary intervention; Peak VO2, peak oxygen uptake; UA, uric acid.


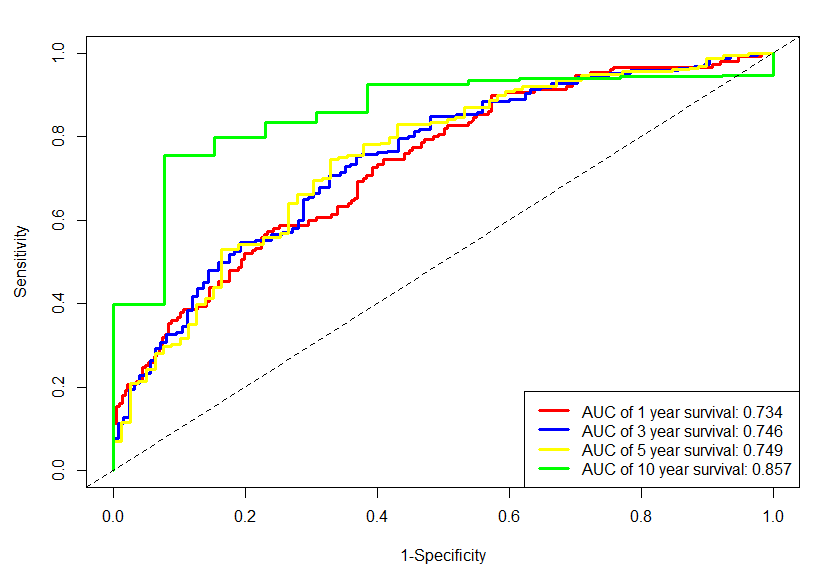


eFigure 3A Derivation group ROC curve


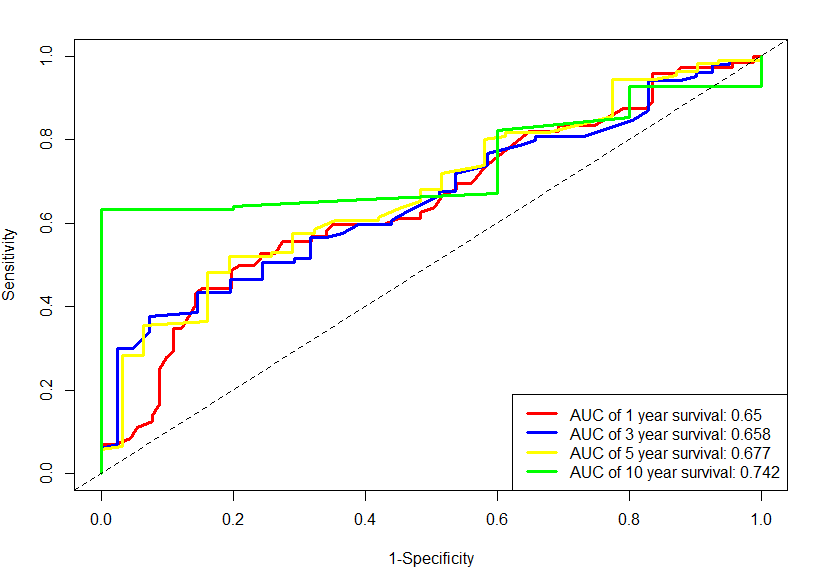


eFigure 3B Validation cohort ROC curve


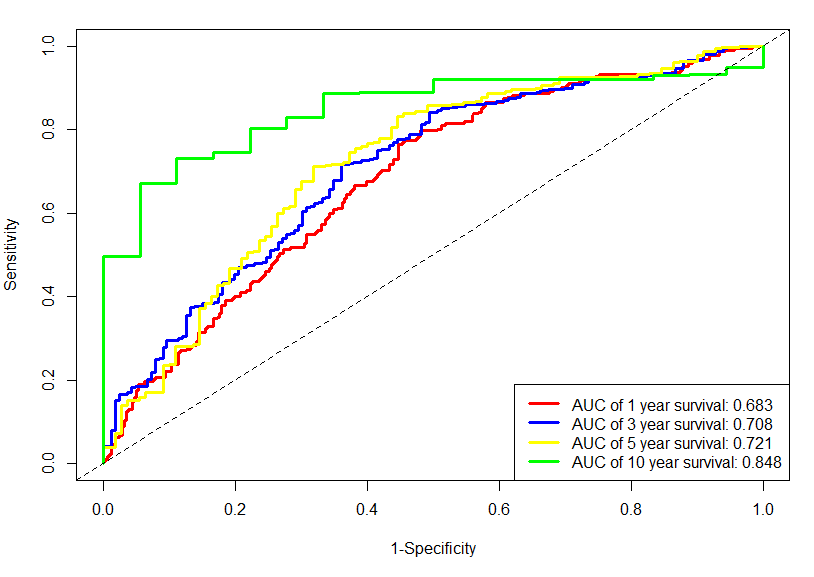


eFigure 3C Full cohort ROC curve

eFigure 3 The area under the receiver-operator characteristic(ROC) curve(AUC) of predicting readmission or death among patients with heart failure.(A)(B)(C)


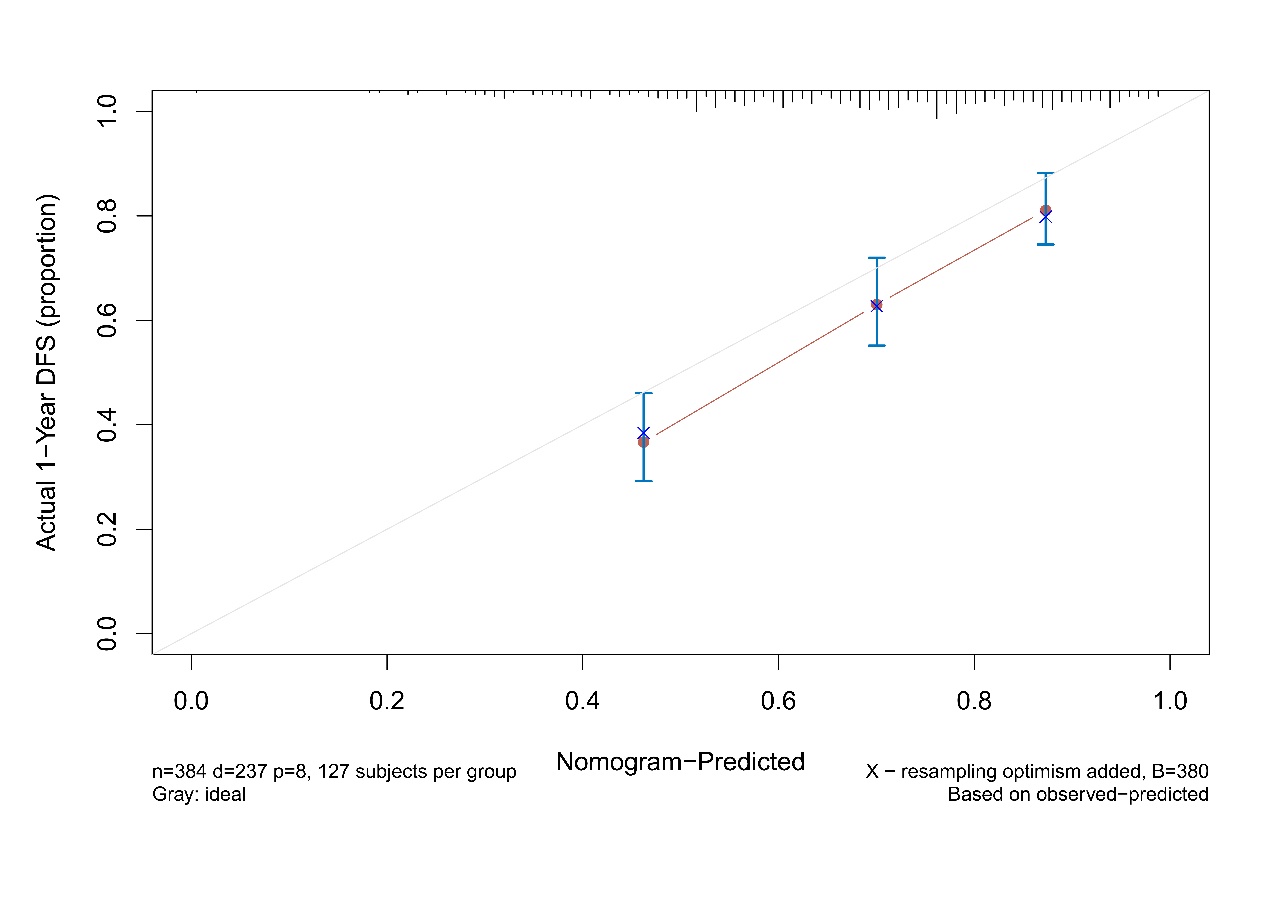


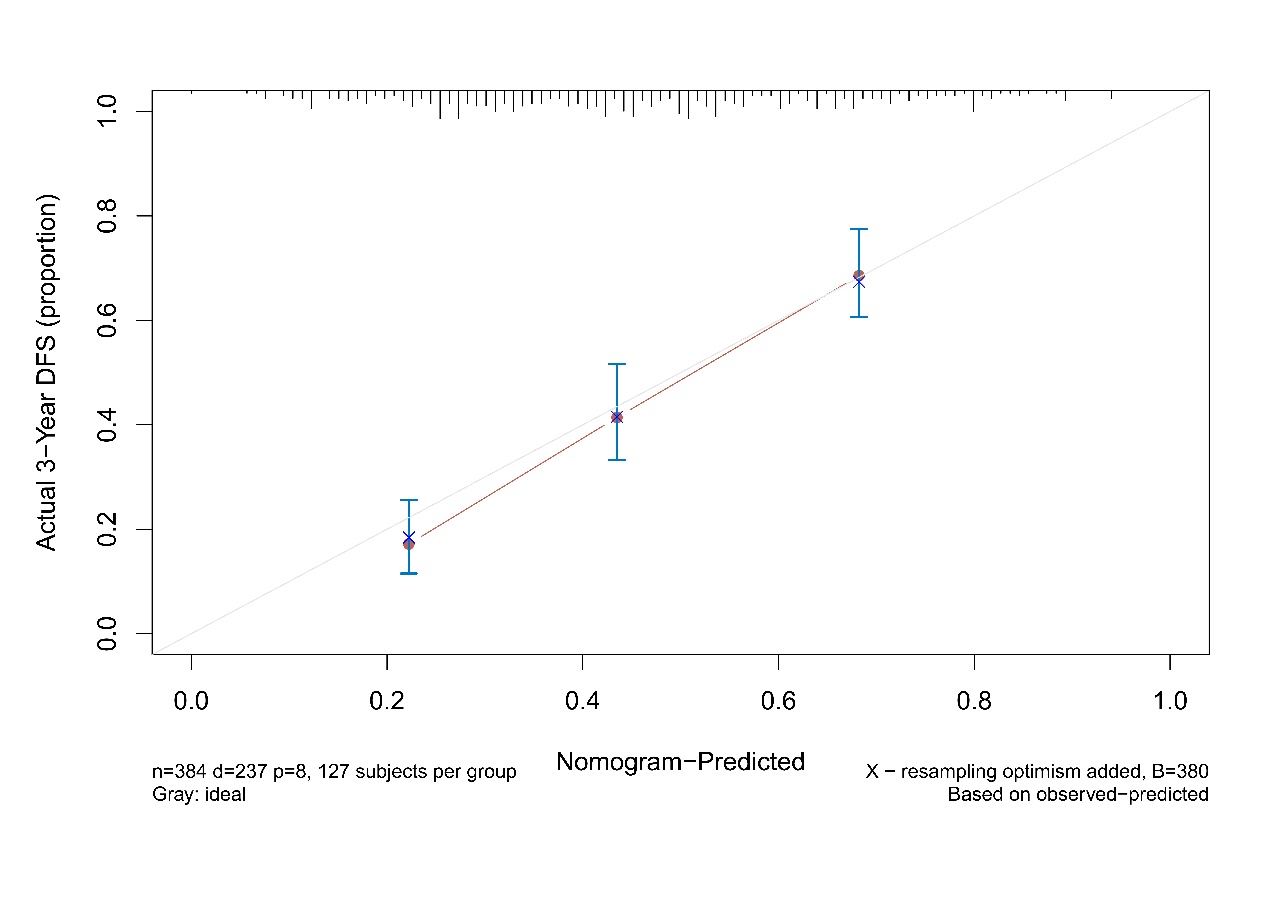


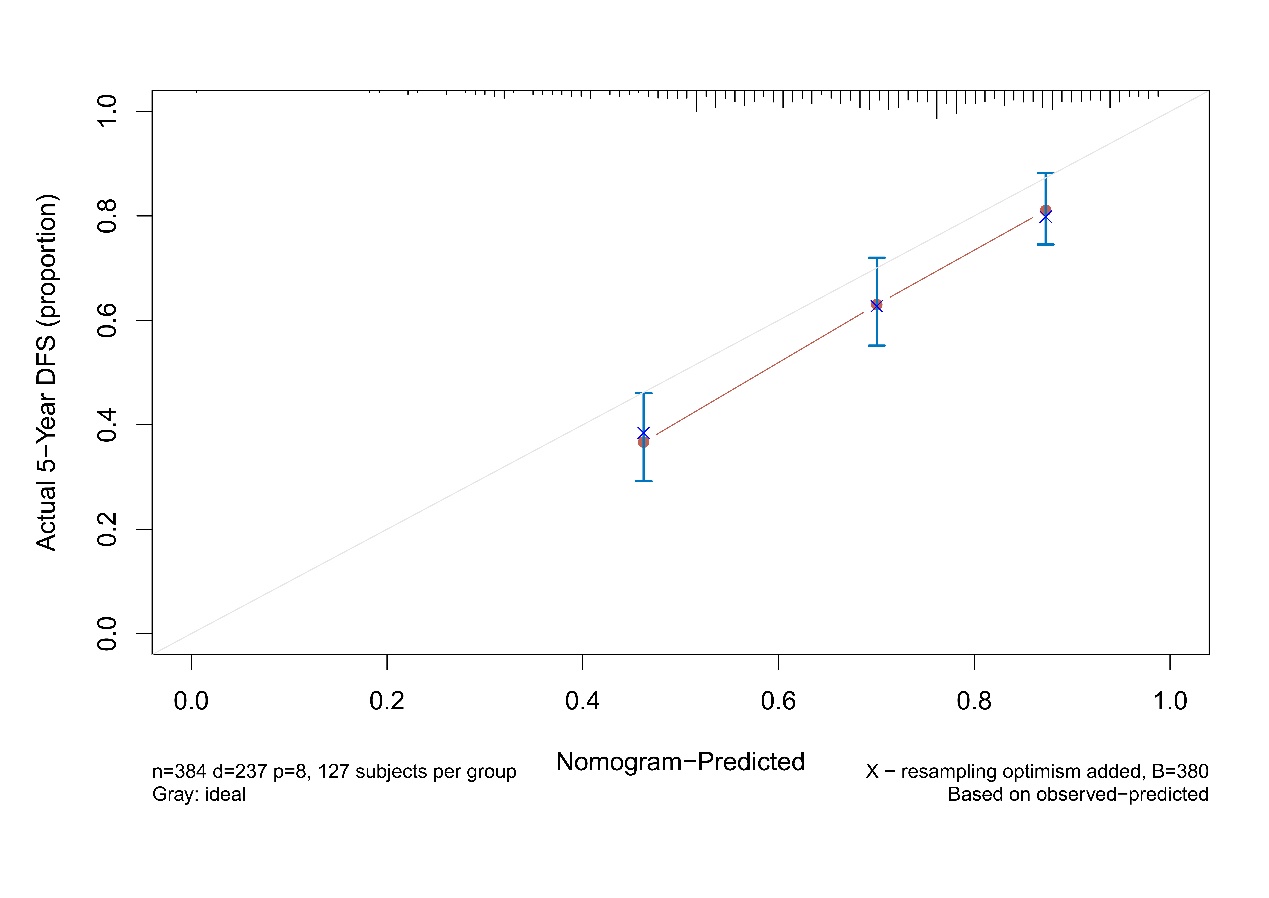


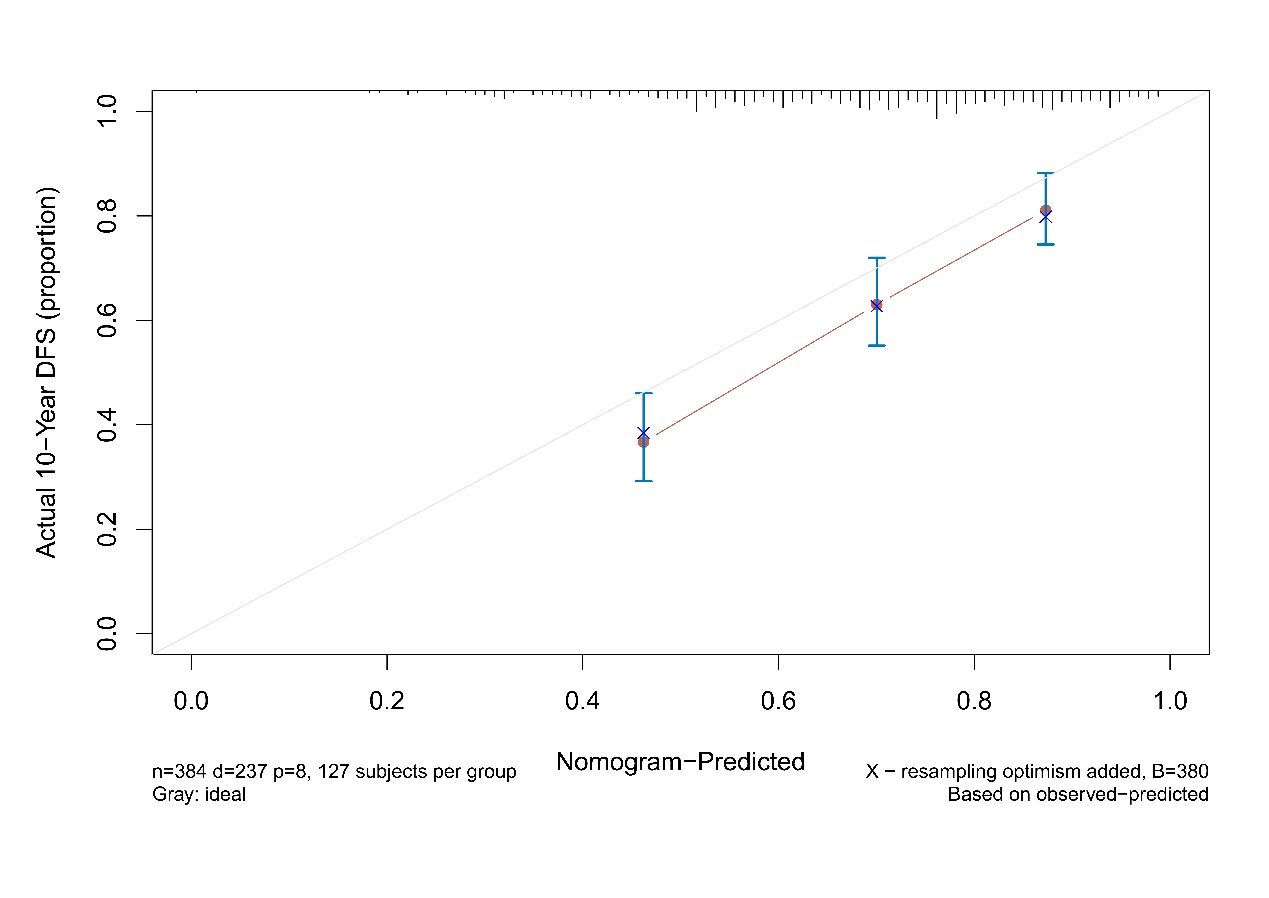


eFigure4A. Derivation group calibration curve.


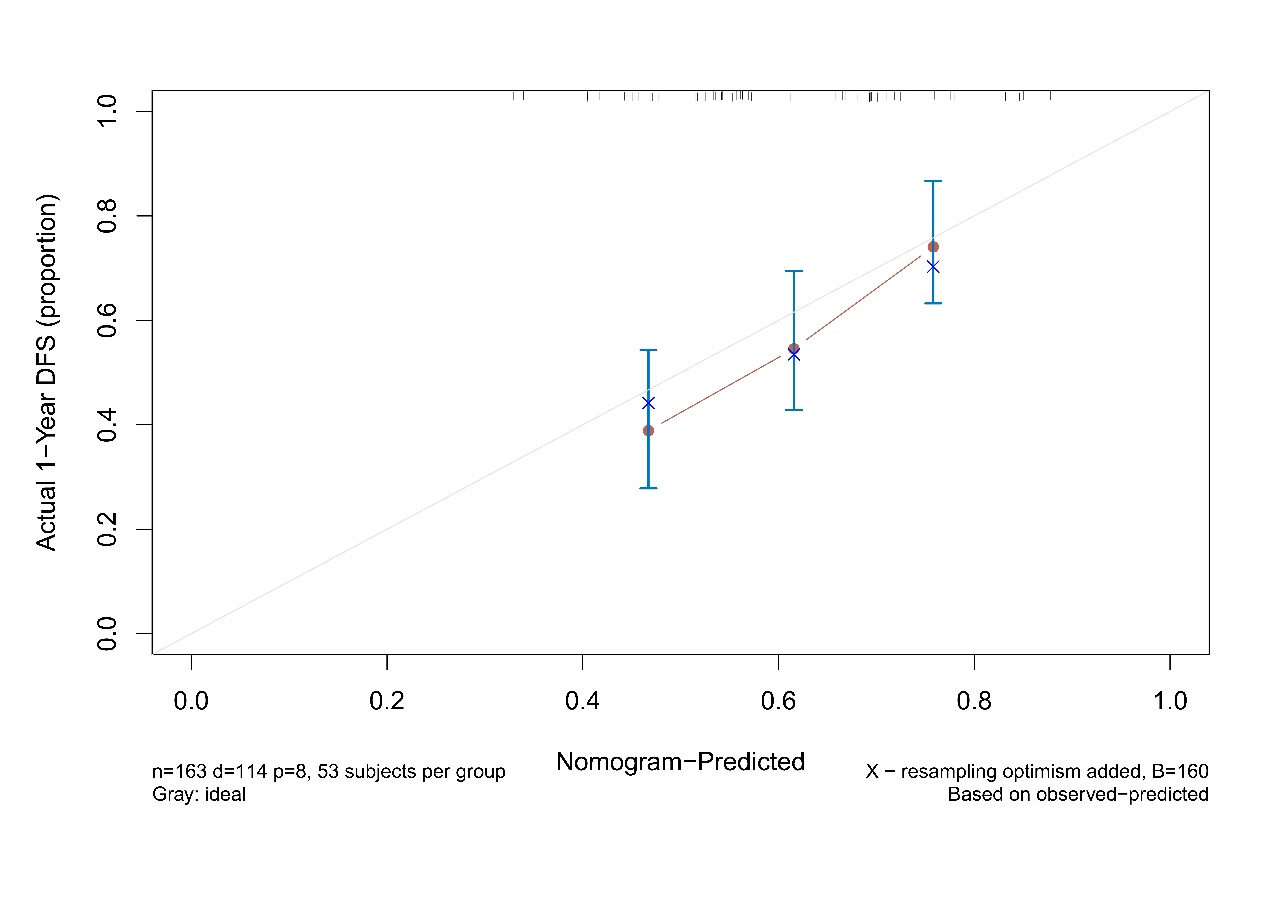


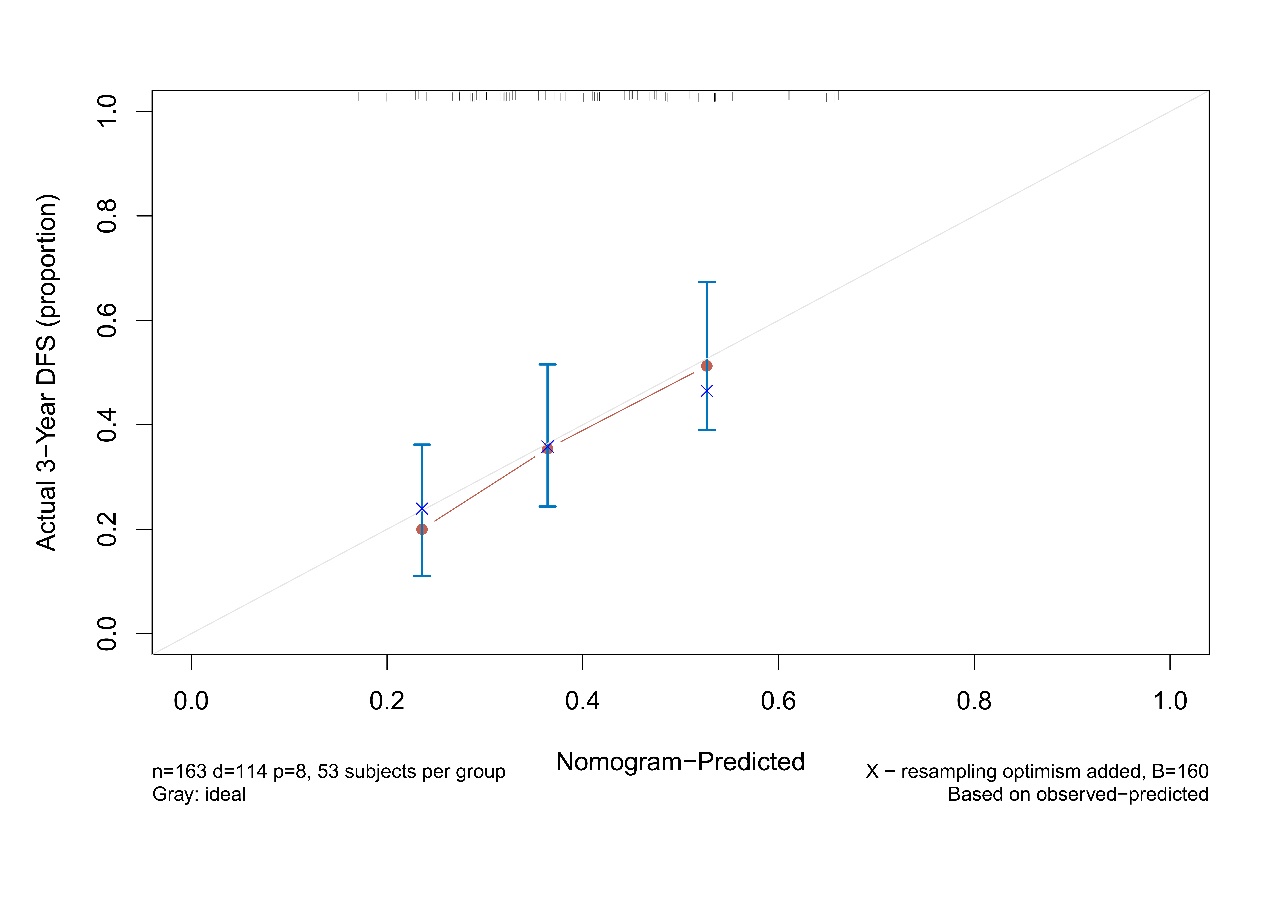


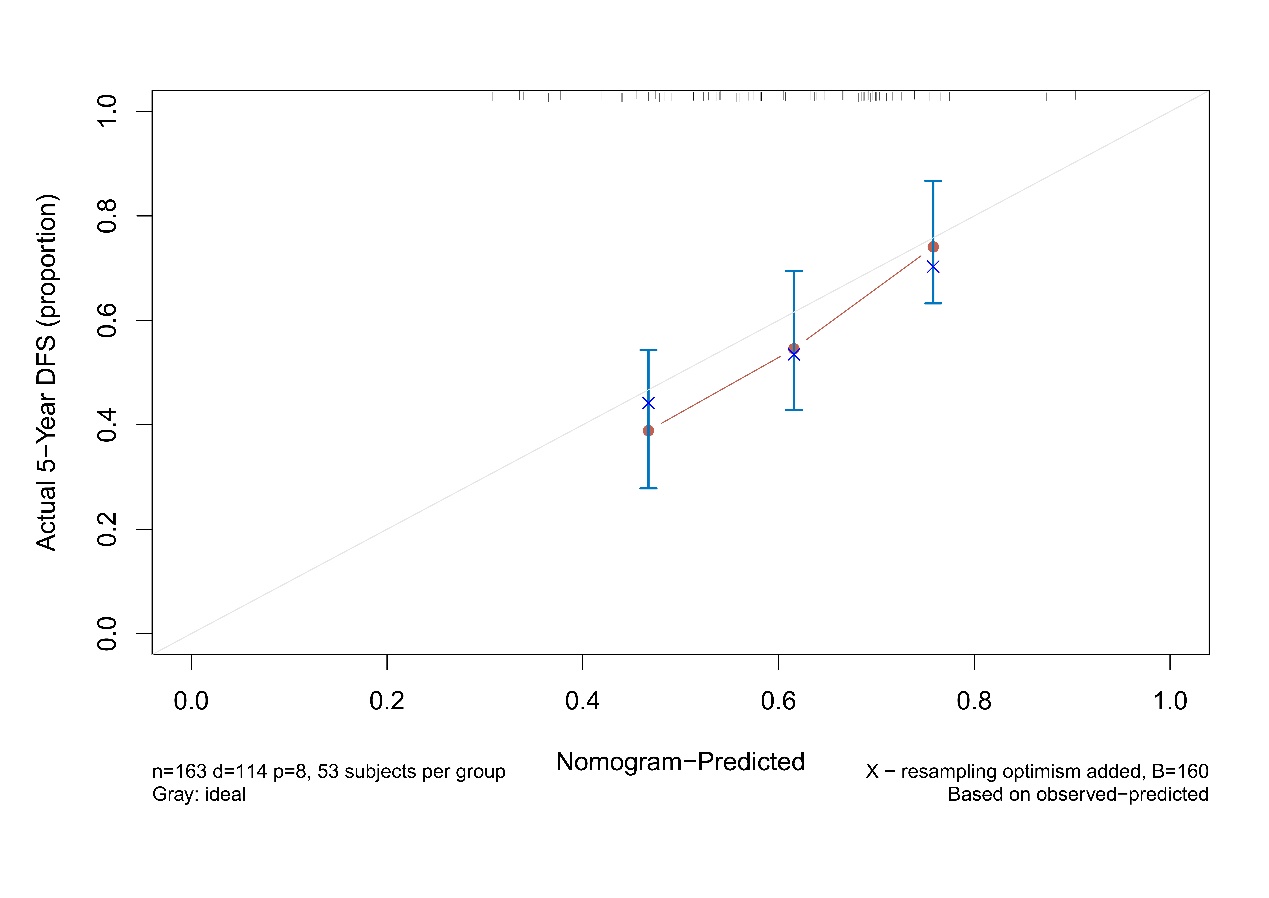


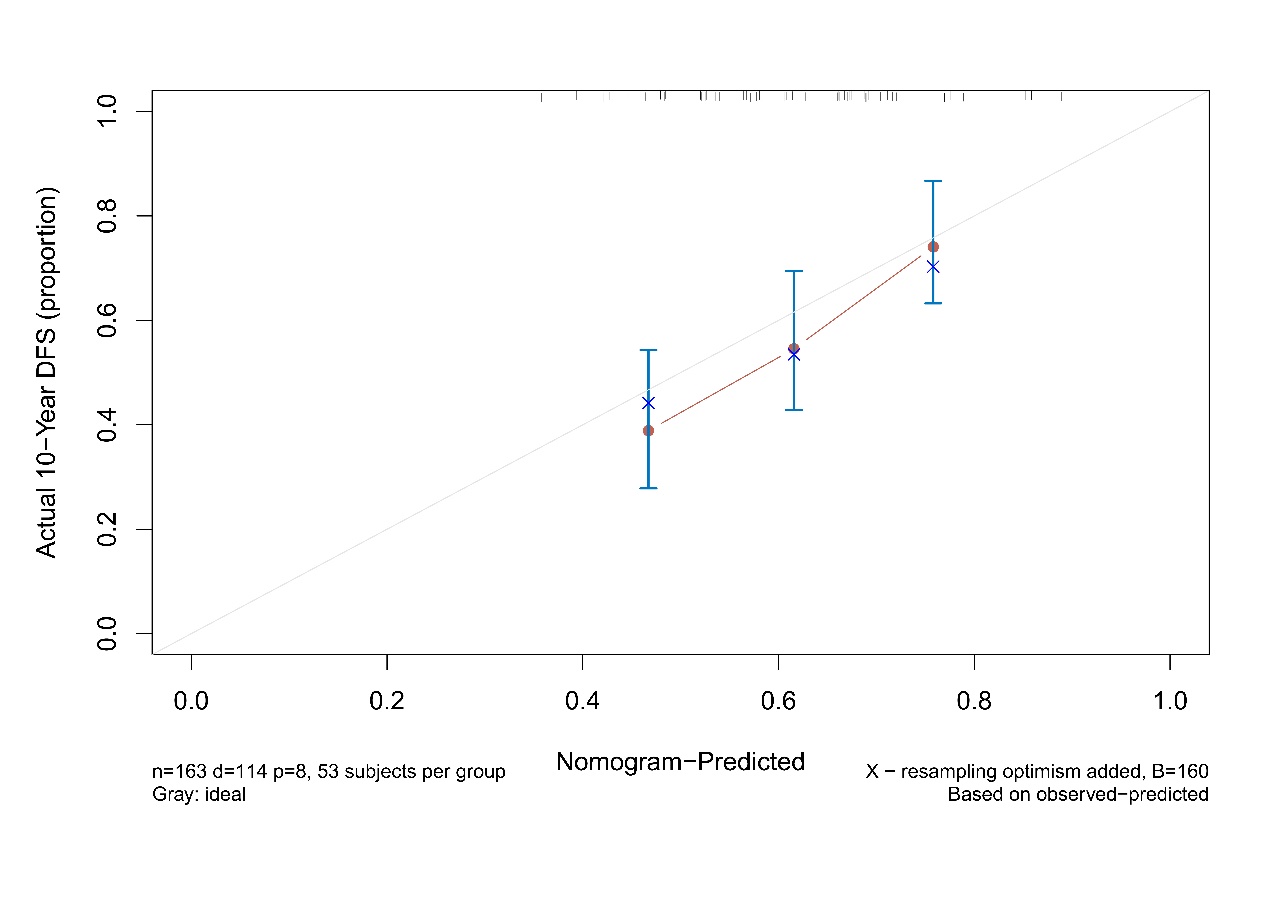


eFigure4B. Validation group calibration curve

eFigure4. Derivation and validation group calibration curve. (A)(B)


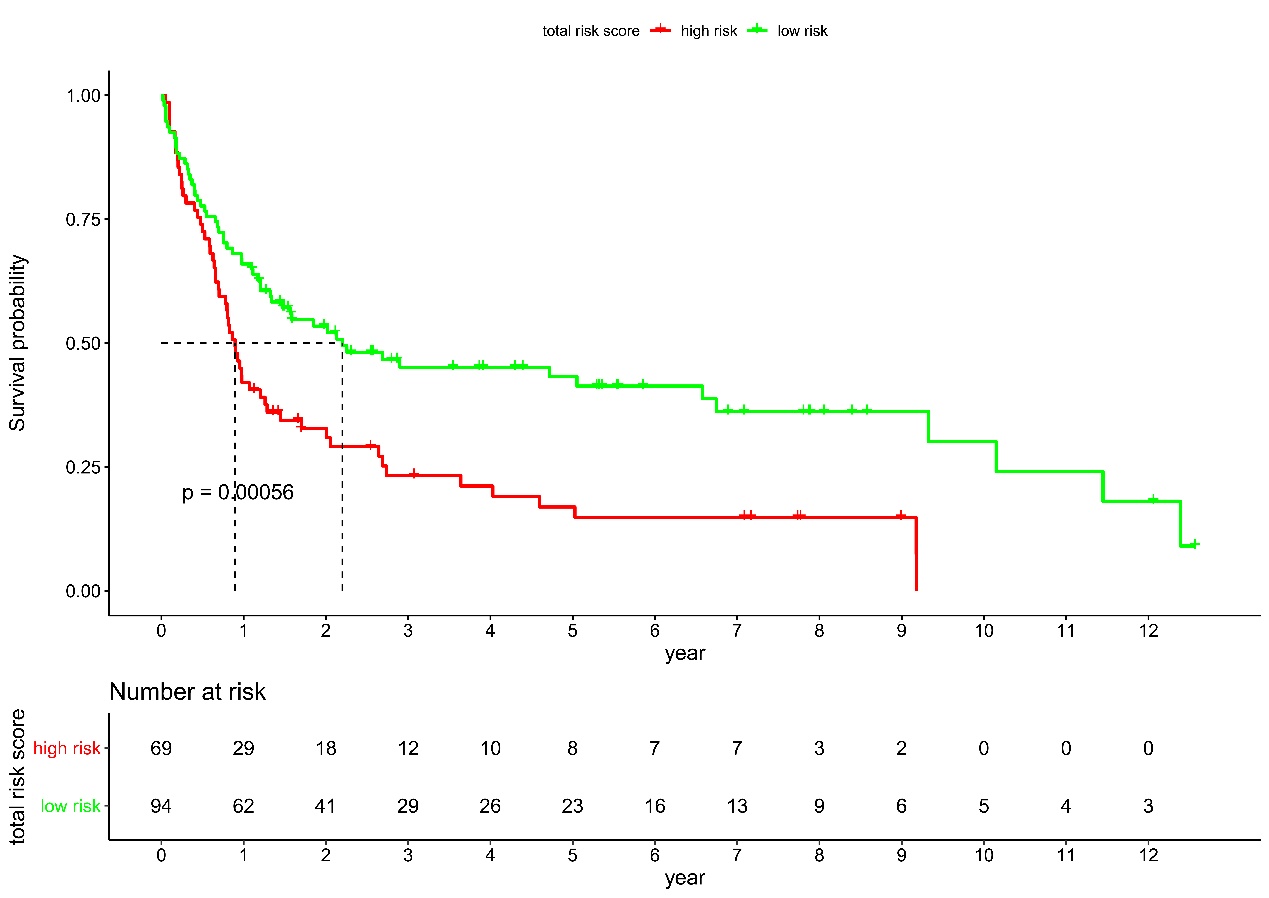


eFigure5A. Validation cohort survival curve


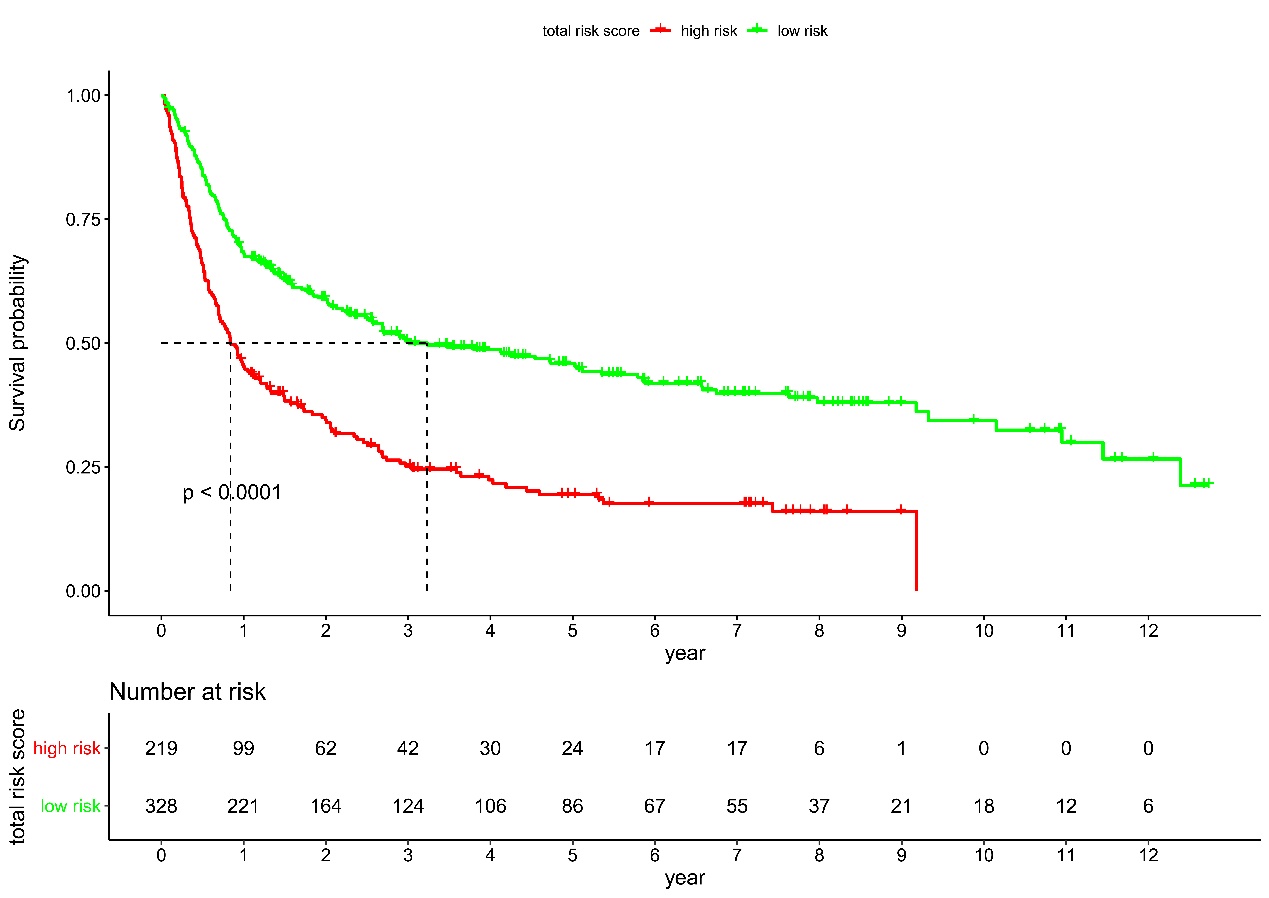


eFigure5B. Full cohort survival curve

eFigure5. Validation and full cohort survival curve. (A)(B)
